# Examining the benefit of graduated compression stockings in the prevention of hospital-associated venous thromboembolism in low-risk surgical patients: a multicentre cluster randomised controlled trial (PETS trial)

**DOI:** 10.1136/bmjopen-2022-069802

**Published:** 2023-01-17

**Authors:** Matthew Machin, Sarrah Peerbux, Sarah Whittley, Beverley J Hunt, Tamara Everington, Manjit Gohel, John Norrie, David Epstein, David J Warwick, Christopher Baker, Zaed Hamady, Sasha Smith, Layla Bolton, Annya Stephens-Boal, Beverley Gray, Joseph Shalhoub, Alun Huw Davies

**Affiliations:** 1 Section of Vascular Surgery, Department of Surgery and Cancer, Charing Cross Hospital, Imperial College London, London, UK; 2 Imperial Vascular Unit, St Mary’s Hospital, Imperial College Healthcare NHS Trust, London, UK; 3 Thrombosis & Haemophilia Centre, Guy’s and St Thomas’ NHS Foundation Trust, London, UK; 4 Hampshire Hospitals NHS Foundation Trust, Winchester, Hampshire, UK; 5 Department of Vascular Surgery, Addenbrooke's Hospital, Cambridge, UK; 6 Usher Institute of Population Health Sciences and Informatics, Edinburgh Clinical Trials Unit, University of Edinburgh, Edinburgh, UK; 7 Faculty of Economic and Business Sciences, University of Granada, Granada, Spain; 8 University Hospital Southampton NHS Foundation Trust, Southampton, UK; 9 General Surgery, University Hospital Southampton NHS Foundation Trust, Southampton, UK

**Keywords:** thromboembolism, surgery, vascular medicine

## Abstract

**Introduction:**

Hospital-acquired thrombosis (HAT) is defined as any venous thromboembolism (VTE)-related event during a hospital admission or occurring up to 90 days post discharge, and is associated with significant morbidity, mortality and healthcare-associated costs. Although surgery is an established risk factor for VTE, operations with a short hospital stay (<48 hours) and that permit early ambulation are associated with a low risk of VTE. Many patients undergoing short-stay surgical procedures and who are at low risk of VTE are treated with graduated compression stockings (GCS). However, evidence for the use of GCS in VTE prevention for this cohort is poor.

**Methods and analysis:**

A multicentre, cluster randomised controlled trial which aims to determine whether GCS are superior in comparison to no GCS in the prevention of VTE for surgical patients undergoing short-stay procedures assessed to be at low risk of VTE. A total of 50 sites (21 472 participants) will be randomised to either intervention (GCS) or control (no GCS). Adult participants (18–59 years) who undergo short-stay surgical procedures and are assessed as low risk of VTE will be included in the study. Participants will provide consent to be contacted for follow-up at 7-days and 90-days postsurgical procedure. The primary outcome is the rate of symptomatic VTE, that is, deep vein thrombosis or pulmonary embolism during admission or within 90 days. Secondary outcomes include healthcare costs and changes in quality of life. The main analysis will be according to the intention-to-treat principle and will compare the rates of VTE at 90 days, measured at an individual level, using hierarchical (multilevel) logistic regression.

**Ethics and dissemination:**

Ethical approval was granted by the Camden and Kings Cross Research Ethics Committee (22/LO/0390). Findings will be published in a peer-reviewed journal and presented at national and international conferences.

**Trial registration number:**

ISRCTN13908683.

**Strengths and limitations of this study**

- This is the first randomised controlled trial evaluating the role of graduated compression stockings (GCS) in the prevention of hospital-associated venous thromboembolism (VTE) in short-stay surgical patients who are assessed as low risk of developing hospital-associated VTE.
- The primary outcome is clinically meaningful and objective in nature.
- The cost-effectiveness analysis will assess the economic implications of providing GCS for the prevention of VTE in low risk, short-stay surgical patients.
- Given this is a cluster design, it will be important to demonstrate that the populations are balanced in terms of confounding factors that is, that the clustering has been successful.

## Introduction

Hospital-associated thrombosis (HAT) is defined as any venous thromboembolism (VTE)-related event during a hospital admission or occurring up to 90 days post discharge 1, and is a term that encompasses both deep vein thrombosis (DVT) and pulmonary embolism (PE). HAT accounts for significant morbidity and mortality with 57.1 VTE-related deaths per 100 000 hospital admissions reported in the year 2018–2019 within the UK National Health Service (NHS).^1^ The probability of untreated moderate-risk surgical inpatients developing HAT was previously estimated to be as high as 15%, this reducing to 4.1% with pharmacological and mechanical prophylaxis.^2^

Previously, UK annual VTE-related mortality was estimated to be 32 000 fatalities if thromboprophylaxis was not used, with associated costs of £640 million per annum.^3^ Surgery is an established risk factor for VTE.^4^ It is estimated that the risk of VTE for patients undergoing most general surgical, urological or open gynaecological procedures without thromboprophylaxis is 10%–40%.^5^ Furthermore, for those undergoing hip or knee arthroplasty, this risk increases to 40%–80%.^5^ Specific surgical factors such as abdominal and pelvic procedures, operations for cancer and procedures with a greater duration are associated with a greater risk of VTE.^6 7^ Conversely, operations with short anaesthetic and procedure times, that can be performed within a <48 hour hospital stay, and that permit early ambulation are associated with a low risk of VTE.^8^

Mechanical thromboprophylaxis in the form of graduated compression stockings (GCS), also known by a brand name ThromboEmbolic Deterrent,^5 9 10^ is often used. GCS apply a level of graduated pressure at rest, that is, not while ambulant; they are designed for immobile patients at risk of VTE. However, it has recently been shown that GCS provide no added benefit in the reduction of HAT for moderate and high VTE risk surgical inpatients receiving prophylactic dose of low-molecular-weight heparin (LMWH).^11^ Additionally, the CLOTS 1 trial, which randomised patients with acute stroke to either standard care alone or the combination of GCS and standard care, reported no difference in the rate of DVT.^12^ The findings of these and other studies have cast doubt on the use of GCS in prevention of VTE. Importantly, the patients in the aforementioned trials were at a higher risk from the distinct low VTE risk group.

Limited evidence is available on the rate of HAT in low-risk surgical patients. In the UK, National Institute for Health and Care Excellence (NICE) guidelines for the prevention of VTE published in 2007 previously recommended that all surgical patients should receive GCS to reduce the risk of VTE, irrespective of the absence of thrombosis risk factors.^13^ The subsequent updated NICE guidelines did not include this blanket recommendation, but instead provided specific recommendations for differing procedure types. The most contemporary NICE guidelines, published in 2018, recommend that all patients undergoing abdominal, thoracic, spinal, bariatric, head and neck and elective joint surgery should be treated with GCS, and to consider treatment with GCS for all those undergoing cardiac, vascular and ear, nose and throat surgery.^14^ The interpretation of these recommendations has meant that patients undergoing short-stay procedures, who are able to ambulate early and have no other thrombosis risk factors are still treated with GCS. Hence, it is now common practice for patients undergoing day case procedures, even those that have no other assessed risk factors, to be prescribed GCS in the absence of contraindications.

The evidence to support GCS for short-stay ambulant patients is poor. A systematic review^15^ examining the use of GCS in comparison to no prophylaxis in low VTE risk short-stay surgical patients confirmed that there have been no randomised controlled trials (RCTs) to support this practice. Furthermore, a meta-analysis in the 2018 Cochrane Review for patients at moderate and high-risk of VTE revealed a significantly lower OR of DVT in those treated with GCS in comparison to those that were not.^16^ However, none of the included 18 surgical RCTs identified in the review consisted of low-risk procedures, that is, ambulatory day case procedures.

Moreover, the UK National Institute for Health Research Health Technology Assessment-funded GAPS trial, assessing the use of GCS in addition to LMWH for the prevention of VTE in moderate and high-risk (ie, not low risk) elective surgical patients, demonstrating that LMWH alone was non-inferior to dual thromboprophylaxis with LMWH and GCS, has further drawn the role of GCS in the prevention of VTE in surgery into question.^11^ This is on the background of other RCTs, such as the CLOTS 1 trial which randomised patients with acute stroke to either standard care or the combination of GCS and standard care and reported no difference in the rate of DVT.^12^ While the clinical community are adopting the GAPS trial findings into clinical practice, they cannot safely be extrapolated to this large cohort of low VTE risk patients who do not also receive LMWH.

Hence, due to this paucity of evidence to support the use of GCS in the prevention of VTE for low VTE risk surgical patients, an adequately powered trial is required to provide evidence in relation to this practice.

### Objectives

The primary objective is to evaluate the potential benefit of GCS in the prevention of HAT in patients undergoing short-stay surgical procedures, assessed as being at low risk for VTE. Secondary objectives include comparisons of quality of life (QoL) at 7-days and 90-days, mortality at 90-days and a cost-effectiveness analysis.

## Methods and analysis

### Trial design

This is a prospective, multicentre, cluster RCT with a follow-up of 3 months. The primary outcome is assessed blindly.

### Study setting

Eligible participants will be recruited from at least 50 NHS Trusts/Health Boards sites in the UK. A complete list of actively recruiting study sites can be obtained from https://clinicaltrials.gov/ct2/show/NCT05347550.

### Eligibility criteria

Inclusion criteria are adult patients aged 18–59 years of age (as age 60 scores 1 point on risk assessment) scheduled to undergo a surgical procedure with a hospital stay <48 hours who are assessed as being at low risk of developing VTE as per the Department of Health risk assessment tool (ie, no assessed thrombosis risk factors/scoring 0).^17^ Exclusion criteria include individuals with a contraindication to GCS; assessed as being at moderate or high-risk of VTE as per the Department of Health risk assessment tool; requiring therapeutic anticoagulation; with thrombophilia or thrombotic disorders; with a previous history of VTE; requiring intermittent pneumatic compression therapy beyond theatre and recovery; requiring extended thromboprophylaxis beyond discharge; female of childbearing age who have a positive pregnancy test; those with lower limb immobilisation; those unable to provide informed consent.

### Interventions

Centres randomised to the intervention arm, which is the current standard of care, will consist of participants receiving GCS. Clinical staff (eg, theatre support workers) will issue stockings to all patients who are scheduled to undergo short-stay surgery. Participants will be instructed to wear their stockings just before undergoing the surgical procedure and to remove the stockings as soon as they are ambulant after the procedure.

In those centres randomised to the control arm, participants will not receive GCS.

**Figure 1:**
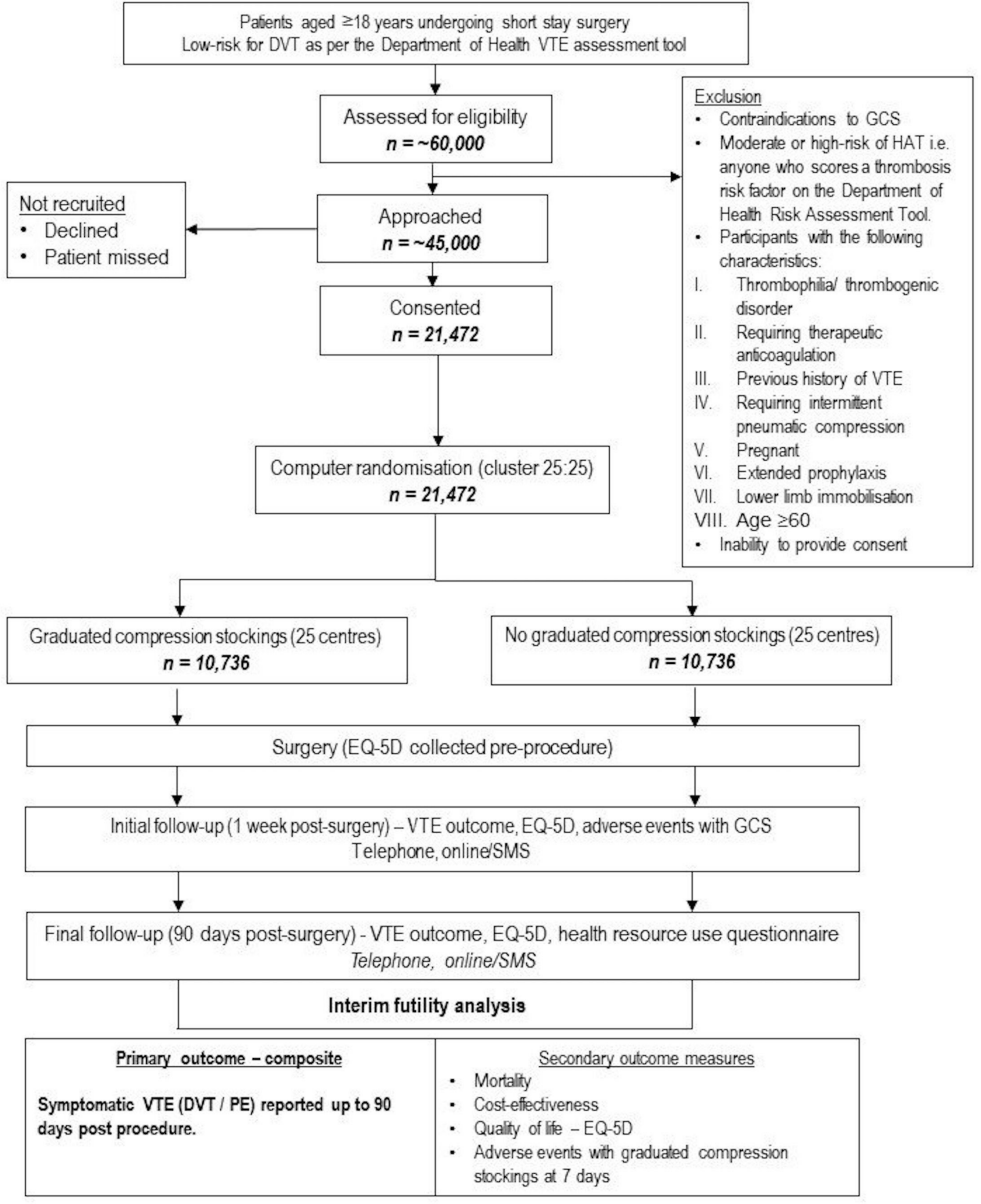
PETS study flow chart. DVT, deep vein thrombosis; EQ-5D, EuroQol-5 Dimension; GCS, graduated compression stockings; HAT, hospital-acquired thrombosis; PE, pulmonary embolism; VTE, venous thromboembolism.

Figure 1 is a diagram displaying the participant flow in the trial.

### Primary outcome

The primary outcome is the rate of symptomatic VTE during admission and/or within 90 days after discharge for surgical patients undergoing short-stay procedures assessed as being at low risk of VTE, in those treated with or without GCS.

### Secondary outcomes

The secondary outcomes include:

1. Mortality rates in each group.
2. Adverse events (AEs) related to the GCS (assessed at 7 days, in those enrolled in the intervention clusters only).
3. QoL score at 7-days and 90-days postsurgical procedure assessed using the EuroQol-5 Dimension(EQ-5D).
4. Resource use and costs of healthcare, and days off work and normal activities, up to 90 days.

### Sample size and study duration

Good quality data for the incidence of VTE in low VTE risk surgical patients not receiving thromboprophylaxis is lacking. Data from the recently published GAPS RCT on moderate and high risk patients reported 29 (1.6%)/1858 VTE events at 90 days.^11^ The VTE rate for short-stay procedures in individuals formally assessed as being at low risk of VTE would be expected to be lower than this, however, as these short-stay patients are not receiving a thromboprophylactic dose of LMWH, we have conservatively assumed an overall control (no stockings nor drugs) VTE rate of 1.0%.

The sample size calculation is for a superiority comparison based on symptomatic VTE (DVT or PE) at 90 days at 90% power with a significance level of 5%, assuming 1.0% in the no stocking (control) and 0.5% in the stocking (active) groups.

Assuming an intraclass correlation coefficient (ICC) on the VTE at 90 days outcome of 0.001 (consistent with the control outcome rate varying uniformly between 0.5% and 1.5%)^18^ and a coefficient of variation of site size of 0.25 (consistent with sites varying according to a normal distribution mean=350 and SD=88, ie 95% of the sites would be between 174 and 526 participants), with 25 sites randomised to active and 25 sites to control, the study would need to recruit 17 500 patients. Allowing for 15% loss to follow, but no sites lost to follow-up, and adjusting for the group sequential design with one formal interim analysis at 50% with 90-day follow-up, 21 472 participants are required. We do not believe there will be any meaningful crossover. A check on the sample size assumptions will be incorporated when there are 10 sites across both arms that have recruited at least 100 patients each (checking on variability in the control outcome for ICC and checking zero crossover). At this stage, as well as confirming the overall maximum sample size, the schedule for a single efficacy or futility analysis at around 50% of the target recruitment with mature 90-day follow-up will be included.

### Interim analysis

When 50% mature primary outcome data are available, a formal interim analysis will be performed with the possibility of stopping early for futility (no prospect of a clinically meaningful treatment effect).

### Recruitment and randomisation

This is a cluster study, hence randomisation will be conducted at recruitment centre level rather than on an individual participant level. Each site will confirm capacity and capability with their local R&D team for participation in the trial prior to randomisation. Sites will be randomised by the Edinburgh Clinical Trials Unit by simple randomisation (1:1) via the Research Electronic Data Capture (REDCap) database, and will be adjusted for based on region, size and VTE compliance within the Trust. The trial manager will be informed of the treatment allocation via email, and the Trust will be informed via a treatment allocation letter. Sites and the study team will undertake local engagement to identify appropriate and willing specialties which form the basic centre unit. Recruiting centres will be randomised/allocated to either the intervention arm (where participants who undergo short-stay surgery will receive GCS to wear during their short-stay procedure) or the control arm (where no GCS will be provided to those undergoing short-stay surgery).

Due to the nature of this cluster design, patients are only required to consent to be contacted for follow-up (at 7- and 90-days post procedure).

Patients from a variety of surgical specialties will be included in this pragmatic trial. Adults who are scheduled to undergo short-stay surgery will be prescreened by a member of the direct care team and invited to speak to a member of the research team. Permission from the patient will be granted by the direct care team before any information is passed or approach made by the research team.

Recruitment will commence on 1 October 2022 for 30 months. The study will close on 31 December 2025.

### Blinding

The primary outcome is assessed blindly. Follow-up data will be collected centrally by blinded assessors at the coordinating centre (Imperial College London).

### Follow-up periods

Participants will be followed up at 7-days and 90-days postsurgical procedure. Follow-up data will be collected via telephone or online questionnaire (the link to the questionnaire will be sent via email or SMS).

At 7 days postsurgical procedure, data on self-reported VTE outcome will be collected and the EQ-5D will be administered. For those participants who were enrolled in the intervention cluster only (ie, sites which were randomised to issue GCS to short-stay surgical patients), information on AEs related to GCS will also be collected.

At 90-days postsurgical procedure, data on self-reported VTE outcome will be collected, together with the EQ-5D and information on the healthcare resource use.

### Data collection and confidentiality

Participant data will be collected and entered onto the web-based database REDCap by the local research team. Data will be filed for 10 years as per local policy and then deleted. Data will be monitored by the trial manager for quality and completeness, and missing data will be requested from the participating sites, as per the data monitoring plan. The trial manager will only have access to pseudonymised data on REDCap.

### Statistical analysis

All statistical analyses will be governed by a comprehensive statistical analysis plan, authored by the study statisticians and agreed by the independent Trial Steering Committee (TSC).

The main analysis will be according to the intention-to-treat principle and will compare the rates of VTE at 90 days, measured at an individual level, using hierarchical (multilevel) logistic regression, adjusting for any other prespecified strongly prognostic individual baseline covariates and site-level baseline covariates, with site itself as a random effect. This will be performed independently by the Edinburgh Clinical Trials Unit who will have sole access to the final REDCap dataset. The findings will be assessed for robustness against any missing data, first using multiple imputation assuming this data are missing at random and, if appropriate and the data permit, further sensitivity analyses will be attempted under any plausible missing data mechanisms not missing at random. Secondary outcomes will be analysed in a similar fashion with generalised linear models appropriate to the distribution of the outcome. Safety data will be summarised descriptively.

### Internal pilot

There will be an internal pilot of feasibility at the end of 6 months of recruitment (beginning of month 7 to end of month 12 of the overall study timeline). We will start recruiting the (minimum) 50 sites at four sites per month. The 100% recruitment rate to meet the sample size is 20 participants per centre month (accounting for the staggered site set-up). This equates to a target of 1440 participants at the end of the 6-month internal pilot. A stringent criterion of <5% failure to receive allocated intervention is set as a marker of crossover between trial arms. Regarding follow-up, a marker of <15% lost to follow-up would be indicative of green progression. The amber criteria is set at 75%–99% of recruitment. This equates to a minimum of 1080 participants across the 6-month internal pilot period and an associated mean recruitment rate of 15 participants per centre month. We have set an amber criterion for site set-up independent of total recruitment number, hence, even if the total participant number is at 100%, if the number of sites set-up is <24 then this would stimulate a review of the site set-up strategy. Any amber criteria would prompt a review of the trial methodology and recruitment strategy. The red criteria, that is, consideration of stopping the trial early, is set at <75% recruitment and is equivalent to achieving fewer than 1080 participants across the 6-month internal pilot period. An independent threshold for site set-up has been set at <18 sites. The TSC will meet at the end of month 13 (allowing 1 month of processing time after the 6 months of recruitment) with blinded estimation of the rate of VTE for each arm. This will confirm the sample size or lead to a sample size re-estimation.

### Cost-effectiveness analysis

Two health economic analyses will be conducted. The main analyses will be performed from the perspective of the NHS and personal social services, with secondary analyses from a societal perspective.

A within-trial analysis will compare GCS to no thromboprophylaxis over the 90 days of the study. Resource use items associated with treatments in hospital and community care will be collected using case notes and self-completed patient resource use diaries over the 90-day follow-up, and costed using manufacturers’ list prices, previous literature and national reference costs. Days off work and normal activities and other patient-related costs will be collected for a secondary analysis. EQ-5D will be collected at baseline and follow-up, analysed using the NICE approved tariff. Appropriate methods will be used to handle missing data and any relevant subgroups in line with the Statistical analysis plan.

If there are clinically relevant and measurable differences in VTE or QoL between the study arms at 90 days, a Markov (state-transition) decision model will be constructed to compare the Incremental Cost-Effectiveness Ratio (ICER) up to 2 years for GCS versus no GCS. The time horizon of the model will be 2 years allowing extrapolation of sequela of VTE events (such as post-thrombotic syndrome (PTS)) over the longer term to quantify the impact of VTE on patient health (quality adjusted life years (QALYs)) and resource use. A preliminary model has been constructed based on published literature to identify the key variables that would need to be collected during the clinical study, and to estimate the number needed to treat (NNT) to avoid one VTE, above which GCS would not be considered cost-effective at NICE thresholds. The 2-year time point was chosen as we know from previous research that the incidence of PTS after acute DVT levels out after the first year.

This preliminary model conservatively assumes 30% of patients with VTE develop PTS, with 3% of those patients having severe PTS. The cost of purchasing and applying GCS stockings is approximately £22.46.^19^ The model assumes the cost of treatment of VTE, non-severe and severe PTS, are £451, £872 and £1547, respectively, and estimates of the utility decrement associated with symptomatic VTE and PTS which are 0.8628, 0.7745 and 0.6752, respectively.^20^ Using a 2-year time horizon, the ICER of GCS versus no prophylaxis would be £20 603 per QALY if the NNT were 200 participants. Hence, for GCS to be cost-effective at a NICE willingness to pay threshold, the NNT would need to be below 200.

The health economic analyses will be conducted and reported according to NICE reference case and Consolidated Health Economic Evaluation Reporting Standards (CHEERS) guidelines,^21 22^ including sensitivity analyses and probabilistic sensitivity analyses. The results will be presented as estimates of mean incremental costs, effects and incremental cost per QALY.

### Data monitoring, safety and quality control

An independent TSC and independent Data Monitoring Committee (iDMC) have been appointed. The main role of the TSC will be to provide overall supervision of the trial and ensure that it is being conducted in accordance with the principles of Good Clinical Practice and the relevant regulations. The main role of the iDMC will be to safeguard the interests of trial participants and to monitor the main outcome measures including safety and efficacy. The clinical trial manager, together with the Trial Management Group, will oversee trial progress.

All treatment-related AEs (related to the GCS only) will be collected centrally at 7 days postprocedure, as will all serious adverse events (SAEs). The Chief Investigator (CI) will be notified of all SAEs within 24 hours. All SAEs will be reported to the Research Ethics Committee (REC) and sponsor if, in the opinion of the CI, the event was related to the intervention. These analyses will be descriptive, with any p values calculated to be interpreted descriptively.

### Patient and public involvement

During the grant application stage, online surveys were launched to learn public views on the PETS study. These surveys helped to inform important aspects of the trial, including the number of visits and questionnaires used in the study. Two patient advisors, both with lived experience of VTE, have been involved in the design of the study and review of patient-facing materials. Both were grant coapplicants, have agreed to sit on the TSC, facilitate patient advisory groups, will assist with the dissemination of results and are coauthors of this manuscript.

### Ethics and dissemination

Ethical approval was granted by the Camden and Kings Cross Research Ethics Committee (22/LO/0390). Amendments to the protocol will be updated on the ISRCTN record. All amendments to the protocol will be submitted to the sponsor for review before applying for approval from the REC and the Health Research Authority (HRA). Protocol amendments will be circulated by email to investigators and study nurses. Standard informed consent will be obtained by research nurses and investigators, with freedom to withdraw at any time. The findings from this study will be published in peer-reviewed journals, presented at national and international conferences and disseminated to participants (via emails and letters at the end of the study).

## Supplementary Material

Reviewer comments

Author's manuscript
