# Supplementary material for: Examining the benefit of graduated compression stockings in the prevention of hospital-associated venous thromboembolism in low-risk surgical patients: a multicentre cluster randomised controlled trial (PETS trial)
Source: BMJ Open. 2023 Jan 17;13(1):e069802. doi: 10.1136/bmjopen-2022-069802 (PMC9853211; doi:10.1136/bmjopen-2022-069802)
Supplement: Author's manuscript [file bmjopen-2022-069802.draft_revisions.pdf]

BMJ Open is committed to open peer review. As part of this commitment we make the peer review history of every article we publish publicly available.

When an article is published we post the peer reviewers' comments and the authors' responses online. We also post the versions of the paper that were used during peer review. These are the versions that the peer review comments apply to.

The versions of the paper that follow are the versions that were submitted during the peer review process. They are not the versions of record or the final published versions. They should not be cited or distributed as the published version of this manuscript.

BMJ Open is an open access journal and the full, final, typeset and author-corrected version of record of the manuscript is available on our site with no access controls, subscription charges or pay-per-view fees (http://bmjopen.bmj.com).

If you have any questions on BMJ Open's open peer review process please email info.bmjopen@bmj.com

BMJ Open

Examining the benefit of graduated compression stockings in the prevention of hospital-associated venous thromboembolism in low-risk surgical patients: a multicentre cluster randomised controlled trial (PETS Trial)

Journal:	*BMJ Open*
Manuscript ID	bmjopen-2022-069802
Article Type:	Protocol
Date Submitted by the Author:	08-Nov-2022
Complete List of Authors:	Machin, Matthew; Imperial College London, Section of Vascular Surgery, Department of Surgery and Cancer, Charing Cross Hospital; Imperial College Healthcare NHS Trust, Imperial Vascular Unit, St Mary's Hospital<br>Peerbux, Sarrah; Imperial College London, Section of Vascular Surgery, Department of Surgery and Cancer, Charing Cross Hospital<br>Whittley, Sarah; Imperial College London, Section of Vascular Surgery, Department of Surgery and Cancer, Charing Cross Hospital<br>Hunt, Beverley; Guy's and St Thomas' NHS Foundation Trust, Thrombosis & Haemophilia Centre<br>Everington, Tamara; Hampshire Hospitals NHS Foundation Trust<br>Gohel, Manjit; Addenbrooke's Hospital, Department of Vascular Surgery<br>Norrie, John; Edinburgh Clinical Trials Unit, University of Edinburgh No. 9, Bioquarter, Usher Institute of Population Health Sciences and Informatics<br>Epstein, David; University of Granada, Faculty of Economic and Business Sciences<br>Warwick, David; University Hospital Southampton NHS Foundation Trust<br>Baker, Christopher; Imperial College Healthcare NHS Trust, Imperial Vascular Unit, St Mary's Hospital<br>Hamady, Zaed ; University Hospital Southampton NHS Foundation Trust, General surgery<br>Smith, Sasha; Imperial College London, Section of Vascular Surgery, Department of Surgery and Cancer, Charing Cross Hospital<br>Bolton, Layla; Imperial College Healthcare NHS Trust, Imperial Vascular Unit, St Mary's Hospital<br>Stephens-Boal, Annya; Imperial College London, Section of Vascular Surgery, Department of Surgery and Cancer, Charing Cross Hospital<br>Gray, Beverley; Imperial College London, Section of Vascular Surgery, Department of Surgery and Cancer, Charing Cross Hospital<br>Shalhoub, Joseph; Imperial College London, Section of Vascular Surgery, Department of Surgery and Cancer, Charing Cross Hospital; Imperial College Healthcare NHS Trust, Imperial Vascular Unit, St Mary's Hospital<br>Davies, AH; Imperial College London, Section of Vascular Surgery, Department of Surgery and Cancer, Charing Cross Hospital; Imperial College Healthcare NHS Trust, Imperial Vascular Unit, St Mary's Hospital
Keywords:	Thromboembolism < CARDIOLOGY, SURGERY, VASCULAR MEDICINE

For peer review only - http://bmjopen.bmj.com/site/about/guidelines.xhtml

SCHOLARONE™
Manuscripts

Title: Examining the benefit of graduated compression stockings in the prevention of hospital-associated venous thromboembolism in low-risk surgical patients: a multicentre cluster randomised controlled trial (PETS Trial)

Authors: Matthew Machin[1,2], Sarrah Peerbux[1], Sarah Whittley[1], Beverley J Hunt[3], Tamara Everington[4], Manjit Gohel[5], John Norrie[6], David Epstein[7], David Warwick[8], Christopher Baker[2], Zaed Hamady[8], Sasha Smith[1], Layla Bolton[2], Annya Stephens-Boal[1], Beverley Gray[1], Joseph Shalhoub[1,2], Alun H. Davies[1,2].

[1] Section of Vascular Surgery, Department of Surgery and Cancer, Imperial College London, Charing Cross Hospital, London W6 8RF, United Kingdom.

[2] Imperial Vascular Unit, Imperial College Healthcare NHS Trust, St Mary's Hospital, London W2 1NY, United Kingdom.

[3] Thrombosis & Haemophilia Centre, Guy's and St Thomas' NHS Foundation Trust, Westminster Bridge Road, London SE1 7EH, United Kingdom.

[4] Hampshire Hospitals NHS Foundation Trust, Aldermaston Road, Basingstoke, Hampshire, RG24 9NA, United Kingdom.

[5] Cambridge Vascular Unit, Addenbrookes Hospital, Cambridge University Hospitals NHS Foundation Trust, Hills Road, Cambridge CB2 0QQ, United Kingdom.

[6] Usher Institute of Population Health Sciences and Informatics, Edinburgh Clinical Trials Unit, University of Edinburgh, Level 2, NINE Edinburgh BioQuarter, 9 Little France Road, Edinburgh, EH16 4UX, United Kingdom

[7] Faculty of Economic and Business Sciences, University of Granada, Hospital Real, Avenida del Hospicio, S/N C.P. 18010, Granada, Spain.

[8] University Hospitals Southampton NHS Foundation Trust, Southampton General Hospital, Tremona Rd, Southampton SO16 6YD, United Kingdom.

For peer review only

Professor Alun Davies and Mr Joseph Shalhoub should be considered joint senior author.

Corresponding Author: Professor Alun H. Davies, Professor of Vascular Surgery, Section of Vascular Surgery, Department of Surgery and Cancer, Imperial College London, Charing Cross Hospital, London W6 8RF, United Kingdom. Email: a.h.davies@imperial.ac.uk.

Keywords: venous thromboembolism, graduated compression stockings, deep venous thrombosis, pulmonary embolism, cluster design.

ABSTRACT

Introduction: Hospital-acquired thrombosis (HAT) is defined as any venous thromboembolism (VTE) related event during a hospital admission or occurring up to 90 days post discharge, and is associated with significant morbidity, mortality and healthcare associated costs. Although surgery is an established risk factor for VTE, operations with a short hospital stay (<48 hours) and that permit early ambulation are associated with a low risk of VTE. Many patients undergoing short-stay surgical procedures and who are at low risk of VTE are treated with graduated compression stockings (GCS). However, evidence for the use of GCS in VTE prevention for this cohort is poor.

Methods and analysis: A multicentre, cluster randomised controlled trial which aims to determine whether GCS are superior in comparison to no GCS in the prevention of VTE for surgical patients undergoing short-stay procedures assessed to be at low risk of VTE. A total of 50 sites (21,472 participants) will be randomised to either intervention (GCS) or control (no GCS). Adult participants (18-59 years) who undergo short-stay surgical procedures and are assessed as low risk of VTE will be included in the study. Participants will provide consent to be contacted for follow-up at 7- and 90-days post-surgical procedure. The primary outcome is the rate of symptomatic VTE, i.e. DVT or PE during admission or within 90 days. Secondary outcomes include healthcare costs and changes in quality of life. The main analysis will be according to the intention-to-treat principle and will compare the rates of VTE at 90 days, measured at an individual level, using hierarchical (multilevel) logistic regression.

Ethics and dissemination: Ethical approval was granted by the Camden and Kings Cross Research

For peer review only - http://bmjopen.bmj.com/site/about/guidelines.xhtml

Ethics Committee (22/LO/0390). Findings will be published in a peer-reviewed journal and presented at national and international conferences.

Registration details: ISRCTN13908683.

ARTICLE SUMMARY

Strengths and Limitations of the study

- To our knowledge, this is the first randomised controlled trial to evaluate the role of GCS in patients who are assessed as low risk of developing hospital-associated VTE and who undergo short-stay surgery
- The primary outcome is clinically meaningful and objective in nature
- This study will complement the results of the GAPS study, which evaluated the role of GCS in patients who were assessed as moderate to high risk of developing hospital-associated VTE
- The cost-effectiveness analysis will assess the economic implications of providing GCS for the prevention of VTE in low risk, short-stay surgical patients
- Given this is a cluster design, it will be important to demonstrate that the populations are balanced in terms of confounding factors i.e., that the clustering has been successful

For peer review only

Word count: 3,291

INTRODUCTION

Hospital-associated thrombosis (HAT) is defined as any venous thromboembolism (VTE) related event occurring during admission and/or within 90 days of hospital admission (1) and is a term that encompasses both deep vein thrombosis (DVT) and pulmonary embolism (PE). HAT accounts for significant morbidity and mortality with 57.1 VTE-related deaths per 100,000 hospital admissions reported in the year 2018-2019 within the UK National Health Service (NHS) (1). The probability of untreated moderate-risk surgical inpatients developing HAT was previously estimated to be as high as 15%, this reducing to 4.1% with pharmacological and mechanical prophylaxis (2).

Previously, UK annual VTE-related mortality was estimated to be 32,000 fatalities if thromboprophylaxis was not used, with associated costs of £640 million per annum (3). Surgery is an established risk factor for VTE (4). It is estimated that the risk of VTE for patients undergoing most general surgical, urological or open gynaecological procedures without thromboprophylaxis is 10-40% (5). Furthermore, for those undergoing hip or knee arthroplasty, this risk increases to 40-80% (5). Specific surgical factors such as abdominal and pelvic procedures, operations for cancer, and procedures with a greater duration are associated with a greater risk of VTE (6,7). Conversely, operations with short anaesthetic and procedure times, that can be performed within a <48-hour hospital stay, and that permit early ambulation are associated with a low risk of VTE (8).

Mechanical thromboprophylaxis in the form of graduated compression stockings (GCS), also known by a brand name ThromboEmbolic Deterrent (TED) (5,9,10), is often used. GCS apply a level of graduated pressure at rest, i.e. not whilst ambulant; they are designed for immobile patients at risk of VTE. However, it has recently been shown that GCS provide no added benefit in the reduction of HAT for moderate and high VTE risk surgical inpatients receiving prophylactic dose of low molecular weight heparin (LMWH) (11). Additionally, the CLOTS 1 trial, which randomised acute stroke patients to either standard care alone or the combination of GCS and standard care, reported no difference in the rate of DVT (12). The findings of these and other studies have cast doubt on the use of GCS in prevention of VTE. Importantly, the patients in the aforementioned trials were at a higher risk from the distinct low VTE risk group.

For peer review only - http://bmjopen.bmj.com/site/about/guidelines.xhtml

Limited evidence is available on the rate of HAT in low risk surgical patients. In the UK, National Institute for Health and Care Excellence (NICE) guidelines for the prevention of VTE published in 2007 previously recommended that all surgical patients should receive GCS to reduce the risk of VTE, irrespective of the absence of thrombosis risk factors (13). The subsequent updated NICE guidelines did not include this blanket recommendation, but instead provided specific recommendations for differing procedure types. The most contemporary NICE guidelines, published in 2018, recommend that all patients undergoing abdominal, thoracic, spinal, bariatric, head and neck, and elective joint surgery should be treated with GCS, and to consider treatment with GCS for all those undergoing cardiac, vascular and ear, nose and throat surgery (14). The interpretation of these recommendations has meant that patients undergoing short-stay procedures, who are able to ambulate early and have no other thrombosis risk factors, are still treated with GCS. Hence, it is now common practice for patients undergoing day case procedures, even those that have no other assessed risk factors, to be prescribed GCS in the absence of contraindications.

The evidence to support GCS for short-stay ambulant patients is poor. A systematic review (15) examining the use of GCS in comparison to no prophylaxis in low VTE risk short stay surgical patients confirmed that there have been no randomised controlled trials (RCTs) to support this practice. Furthermore, a meta-analysis in the 2018 Cochrane Review for patients at moderate and high-risk of VTE revealed a significantly lower odds ratio of DVT in those treated with GCS in comparison to those that were not (16). However, none of the included 18 surgical RCTs identified in the review consisted of low risk procedures, i.e. ambulatory day case procedures.

Moreover, the UK National Institute for Health Research (NIHR) Health Technology Assessment (HTA)-funded GAPS trial, assessing the use of GCS in addition to LMWH for the prevention of VTE in moderate and high-risk (i.e. not low risk) elective surgical patients, demonstrating that LMWH alone was non-inferior to dual thromboprophylaxis with LMWH and GCS, has further drawn the role of GCS in the prevention of VTE in surgery into question (11). This is on the background of other RCTs, such as the CLOTS 1 trial which randomised acute stroke patients to either standard care or the combination of GCS and standard care and reported no difference in the rate of DVT (12). While the clinical community are adopting the GAPS trial findings into clinical practice, they cannot safely be extrapolated to this large cohort of low VTE risk patients who don't also receive LMWH.

For peer review only - http://bmjopen.bmj.com/site/about/guidelines.xhtml

Hence, due to this paucity of evidence to support the use of GCS in the prevention of VTE for low VTE risk surgical patients, an adequately powered trial is required to provide evidence in relation to this practice.

Objectives

The primary objective is to evaluate the potential benefit of GCS in the prevention of HAT in patients undergoing short-stay surgical procedures, assessed as being at low risk for VTE. Secondary objectives include comparisons of quality of life (QoL) at 7- and 90-days, mortality at 90-days, and a cost-effectiveness analysis.

METHODS AND ANALYSIS

Trial design

This is a prospective, multicentre, cluster randomised controlled trial with a follow-up of three months. The primary outcome is assessed blindly.

Study Setting

Eligible participants will be recruited from at least 50 NHS Trusts/Health Boards sites in the United Kingdom.

Eligibility Criteria

Inclusion criteria are: adult patients aged 18-59 years of age (as age 60 scores 1 point on risk assessment) scheduled to undergo a surgical procedure with a hospital stay <48 hours who are assessed as being at low risk of developing VTE as per the Department of Health risk assessment tool (i.e. no assessed thrombosis risk factors / scoring 0) (17). Exclusion criteria include individuals: with a contraindication to GCS; assessed as being at moderate or high-risk of VTE as per the Department of Health risk assessment tool; requiring therapeutic anticoagulation; with thrombophilia or thrombotic disorders; with a previous history of VTE; requiring intermittent pneumatic compression therapy beyond theatre and recovery; requiring extended thromboprophylaxis beyond discharge; female of childbearing

For peer review only - http://bmjopen.bmj.com/site/about/guidelines.xhtml

age who have a positive pregnancy test; those with lower limb immobilisation; those unable to provide informed consent.

Interventions

Centres randomised to the intervention arm, which is the current standard of care, will consist of participants receiving GCS. Clinical staff (e.g. theatre support workers) will issue stockings to all patients who are scheduled to undergo short-stay surgery. Participants will be instructed to wear their stockings just before undergoing the surgical procedure and to remove the stockings as soon as they are ambulant after the procedure.

In those centres randomised to the control arm, participants will not receive GCS.

Figure 1 is a diagram displaying the participant flow in the trial.

Primary outcome

The primary outcome is the rate of symptomatic VTE during admission and/or within 90 days after discharge for surgical patients undergoing short-stay procedures assessed as being at low risk of VTE, in those treated with or without GCS.

Secondary outcomes

The secondary outcomes include:

- Mortality rates in each group
- Adverse events related to the GCS (assessed at 7-days, in those enrolled in the intervention clusters only)
- QoL score at 7- and 90-days post-surgical procedure assessed using the EQ-5D
- Resource use and costs of healthcare, and days off work and normal activities, up to 90 days

Sample size and study duration

Good quality data for the incidence of VTE in low VTE risk surgical patients not receiving thromboprophylaxis is lacking. Data from the recently published GAPS RCT on moderate and high risk patients reported 29/1858 (1.6%) VTE events at 90 days (11). The VTE rate for short stay procedures

For peer review only - http://bmjopen.bmj.com/site/about/guidelines.xhtml

in individuals formally assessed as being at low risk of VTE would be expected to be lower than this, however, as these short stay patients are not receiving a thromboprophylactic dose of LMWH, we have conservatively assumed an overall control (no stockings nor drugs) VTE rate of 1.0%.

The sample size calculation is for a superiority comparison based on symptomatic VTE (DVT or PE) at 90 days at 90% power with a significance level of 5%, assuming 1.0% in the no stocking (control) and 0.5% in the stocking (active) groups.

Assuming an intraclass correlation coefficient (ICC) on the VTE at 90 days outcome of 0.001 (consistent with the control outcome rate varying uniformly between 0.5% and 1.5%) (18) and a coefficient of variation of site size of 0.25 (consistent with sites varying according to a Normal distribution mean=350 and SD=88 i.e. 95% of the sites would be between 174 and 526 participants), with 25 sites randomised to active and 25 sites to control, the study would need to recruit 17,500 patients. Allowing for 15% loss to follow, but no sites lost to follow up, and adjusting for the group sequential design with one formal interim analysis at 50% with 90-day follow up, 21,472 participants are required. We do not believe there will be any meaningful crossover. A check on the sample size assumptions will be incorporated when there are 10 sites across both arms that have recruited at least 100 patients each (checking on variability in the control outcome for ICC, and checking zero crossover). At this stage, as well as confirming the overall maximum sample size, the schedule for a single efficacy or futility analysis at around 50% of the target recruitment with mature 90-day follow-up will be included.

Interim analysis

When 50% mature primary outcome data is available, a formal interim analysis will be performed with the possibility of stopping early for futility (no prospect of a clinically meaningful treatment effect).

Recruitment and randomisation

This is a cluster study, hence randomisation will be conducted at recruitment centre level rather than on an individual participant level. Each site will confirm capacity and capability with their local R&D team for participation in the trial prior to randomisation. Sites and the study team will undertake local engagement to identify appropriate and willing specialties which form the basic centre unit. Recruiting centres will be randomised/allocated to either the intervention arm (where participants who undergo

For peer review only

For peer review only - http://bmjopen.bmj.com/site/about/guidelines.xhtml

short-stay surgery will receive GCS to wear during their short-stay procedure) or the control arm (where no GCS will be provided to those undergoing short-stay surgery).

Due to the nature of this cluster design, patients are only required to consent to be contacted for follow-up (at 7- and 90-days post procedure).

Patients from a variety of surgical specialties will be included in this pragmatic trial. Adults who are scheduled to undergo short-stay surgery will be pre-screened by a member of the direct care team and invited to speak to a member of the research team. Permission from the patient will be granted by the direct care team before any information is passed or approach made by the research team.

Recruitment will commence on 01/10/2022 for 30-months. The study will close on 31/12/2025.

Blinding

The primary outcome is assessed blindly. Follow-up data will be collected centrally by the coordinating centre (Imperial College London).

Follow-up periods

Participants will be followed-up by at 7- and 90-days post-surgical procedure. Follow-up data will be collected via telephone or online questionnaire (the link to the questionnaire will be sent via email or SMS).

At 7-days post-surgical procedure, data on self-reported VTE outcome will be collected and the EQ-5D will be administered. For those participants who were enrolled in the intervention cluster only (i.e. sites which were randomised to issue GCS to short-stay surgical patients), information on adverse events related to GCS will also be collected.

At 90-days post-surgical procedure, data on self-reported VTE outcome will be collected, together with the EQ-5D and information on the healthcare resource use.

Data collection and confidentiality

Participant data will be stored in the password-protected Research Electronic Data Capture (REDCap) database. Data will be monitored for quality and completeness, and missing data will be requested from the participating sites, as per the data monitoring plan.

For peer review only - http://bmjopen.bmj.com/site/about/guidelines.xhtml

Statistical analysis

All statistical analyses will be governed by a comprehensive Statistical Analysis Plan, authored by the study statisticians and agreed by the independent Trial Steering Committee (TSC).

The main analysis will be according to the intention-to-treat principle and will compare the rates of VTE at 90 days, measured at an individual level, using hierarchical (multilevel) logistic regression, adjusting for any other pre-specified strongly prognostic individual baseline covariates and site-level baseline covariates, with site itself as a random effect. The findings will be assessed for robustness against any missing data, first using multiple imputation assuming this data is missing at random and, if appropriate and the data permits, further sensitivity analyses will be attempted under any plausible missing data mechanisms not missing at random. Secondary outcomes will be analysed in a similar fashion with generalised linear models appropriate to the distribution of the outcome. Safety data will be summarised descriptively.

Internal pilot

There will be an internal pilot of feasibility at the end of 6 months of recruitment (beginning of month 7 to end of month 12 of the overall study timeline). We will start recruiting the (minimum) 50 sites at four sites per month. The 100% recruitment rate to meet the sample size is 20 participants per centre month (accounting for the staggered site set-up). This equates to a target of 1440 participants at the end of the 6-month internal pilot. A stringent criterion of <5% failure to receive allocated intervention is set as a marker of crossover between trial arms. Regarding follow-up, a marker of <15% lost to follow-up would be indicative of green progression. The amber criteria is set at 75 – 99% of recruitment. This equates to a minimum of 1,080 participants across the 6-month internal pilot period and an associated mean recruitment rate of 15 participants per centre-month. We have set an amber criterion for site set-up independent of total recruitment number, hence, even if the total participant number is at 100%, if the number of sites set-up is <24 then this would stimulate a review of the site set-up strategy. Any amber criteria would prompt a review of the trial methodology and recruitment strategy. The red criteria, i.e. consideration of stopping the trial early, is set at <75% recruitment and is equivalent to achieving fewer than 1,080 participants across the 6-month internal pilot period. An independent threshold for site set-up has been set at <18 sites. The TSC will meet at the end of month 13 (allowing 1 month of

For peer review only - http://bmjopen.bmj.com/site/about/guidelines.xhtml

processing time after the 6 months of recruitment) with blinded estimation of the rate of VTE for each arm. This will confirm the sample size or lead to a sample size re-estimation.

Cost-effectiveness analysis

Two health economic analyses will be conducted. The main analyses will be performed from the perspective of the NHS and Personal Social Services, with secondary analyses from a societal perspective.

A within-trial analysis will compare GCS to no thromboprophylaxis over the 90 days of the study. Resource use items associated with treatments in hospital and community care will be collected using case notes and self-completed patient resource use diaries over the 90-day follow-up, and costed using manufacturers' list prices, previous literature and national reference costs. Days off work and normal activities and other patient-related costs will be collected for a secondary analysis. EQ-5D will be collected at baseline and follow up, analysed using the NICE approved tariff. Appropriate methods will be used to handle missing data and any relevant subgroups in line with the Statistical Analysis Plan.

If there are clinically relevant and measurable differences in VTE or quality of life between the study arms at 90 days, a Markov (state–transition) decision model will be constructed to compare the ICER up to 2 years for GCS versus no GCS. The time horizon of the model will be 2 years allowing extrapolation of sequela of VTE events (such as post-thrombotic syndrome [PTS]) over the longer term to quantify the impact of VTE on patient health (quality adjusted life years [QALYs]) and resource use. A preliminary model has been constructed based on published literature to identify the key variables that would need to be collected during the clinical study, and to estimate the number needed to treat (NNT) to avoid 1 VTE, above which GCS would not be considered cost-effective at NICE thresholds. The 2-year time point was chosen as we know from previous research that the incidence of PTS after acute DVT levels out after the first year.

This preliminary model conservatively assumes 30% of patients with VTE develop PTS, with 3% of those patients having severe PTS. The cost of purchasing and applying GCS stockings is approximately £22.46 (19). The model assumes the cost of treatment of VTE, non-severe and severe PTS, are £451, £872 and £1547, respectively, and estimates of the utility decrement associated with symptomatic VTE and PTS which are 0.8628, 0.7745 and 0.6752, respectively (20). Using a 2-year time horizon, the ICER of GCS versus no prophylaxis would be £20,603 per QALY if the NNT were 200 participants. Hence,

For peer review only - http://bmjopen.bmj.com/site/about/guidelines.xhtml

299 for GCS to be cost-effective at a NICE willingness to pay threshold, the NNT would need to be below 300 200.

301 The health economic analyses will be conducted and reported according to NICE reference case and 302 CHEERS guidelines (21,22), including sensitivity analyses and probabilistic sensitivity analyses. The 303 results will be presented as estimates of mean incremental costs, effects, and incremental cost per 304 QALY.

305 **Data monitoring, safety and quality control**

306 An independent TSC and independent Data Monitoring Committee (iDMC) will be appointed. The main 307 role of the TSC will be to provide overall supervision of the trial and ensure that it is being conducted in 308 accordance with the principles of Good Clinical Practice and the relevant regulations. The main role of 309 the iDMC will be to safeguard the interests of trial participants and to monitor the main outcome 310 measures including safety and efficacy. The clinical trial manager, together with the Trial Management 311 Group, will oversee trial progress.

312 All treatment-related adverse events (AEs; related to the GCS only) will be collected as will all serious 313 adverse events (SAEs). The chief investigator will be notified of all SAEs within 24 hours. All SAEs will 314 be reported to the research ethics committee (REC) if, in the opinion of the CI, the event was related to 315 the intervention. These analyses will be descriptive, with any p-values calculated to be interpreted 316 descriptively.

317 **Patient and public involvement**

318 During the grant application stage, online surveys were launched to learn public views on the PETS 319 study. These surveys helped to inform important aspects of the trial, including the number of visits and 320 questionnaires used in the study. Two patient advisors, both with lived experience of VTE, have been 321 involved in the design of the study and review of patient facing materials. Both were grant co-applicants, 322 have agreed to sit on the Trial Steering Committee, facilitate patient advisory groups, will assist with the 323 dissemination of results, and are co-authors of this manuscript.

324

325 **ETHICS AND DISSEMINATION**

326 Ethical approval was granted by the Camden and Kings Cross Research Ethics Committee

For peer review only - http://bmjopen.bmj.com/site/about/guidelines.xhtml

(22/LO/0390). Amendments to the protocol will be updated on the ISRCTN record. All amendments to the protocol will be submitted to the sponsor for review before applying for approval from the REC and the Health Research Authority (HRA). Standard informed consent will be taken with freedom to withdraw at any time. The findings from this study will be published peer-reviewed journals, presented at national and international conferences and to participants (via emails and letters at the end of the study)

For peer review only

For peer review only - http://bmjopen.bmj.com/site/about/guidelines.xhtml

REFERENCES

1. 5.1 Deaths from venous thromboembolism (VTE) related events within 90 days post discharge from hospital [Internet]. NHS Digital. [cited 2022 Mar 29]. Available from: https://digital.nhs.uk/data-and-information/publications/statistical/nhs-outcomes-framework/march-2022/domain-5---treating-and-caring-for-people-in-a-safe-environment-and-protecting-them-from-avoidable-harm-nof/5.1-deaths-from-venous-thromboembolism-vte-related-events-within-90-days-post-discharge-from-hospital

2. Cohen AT, Agnelli G, Anderson FA, Arcelus JI, Bergqvist D, Brecht JG, et al. Venous thromboembolism (VTE) in Europe. The number of VTE events and associated morbidity and mortality. Thromb Haemost. 2007 Oct;98(4):756–64.

3. The Prevention of Venous Thromboembolism in Hospitalised Patients Second Report of Session 2004-05.

4. Anderson FA, Spencer FA. Risk factors for venous thromboembolism. Circulation. 2003 Jun 17;107(23 Suppl 1):I9-16.

5. Geerts WH, Bergqvist D, Pineo GF, Heit JA, Samama CM, Lassen MR, et al. Prevention of venous thromboembolism: American College of Chest Physicians Evidence-Based Clinical Practice Guidelines (8th Edition). Chest. 2008 Jun;133(6 Suppl):381S-453S.

6. Gould MK, Garcia DA, Wren SM, Karanicolas PJ, Arcelus JI, Heit JA, et al. Prevention of VTE in nonorthopedic surgical patients: Antithrombotic Therapy and Prevention of Thrombosis, 9th ed: American College of Chest Physicians Evidence-Based Clinical Practice Guidelines. Chest. 2012 Feb;141(2 Suppl):e227S-e277S.

7. Sweetland S, Green J, Liu B, Berrington de González A, Canonico M, Reeves G, et al. Duration and magnitude of the postoperative risk of venous thromboembolism in middle aged women: prospective cohort study. BMJ. 2009 Dec 3;339:b4583.

8. Hair A, Duffy K, McLean J, Taylor S, Smith H, Walker A, et al. Groin hernia repair in Scotland. Br J Surg. 2000 Dec;87(12):1722–6.

For peer review only - http://bmjopen.bmj.com/site/about/guidelines.xhtml

9. Overview | Venous thromboembolic diseases: diagnosis, management and thrombophilia testing | Guidance | NICE [Internet]. NICE; [cited 2022 Mar 29]. Available from: https://www.nice.org.uk/guidance/ng158

10. Compression stockings | Health topics A to Z | CKS | NICE [Internet]. [cited 2022 Mar 29]. Available from: https://cks.nice.org.uk/topics/compression-stockings/

11. Shalhoub J, Lawton R, Hudson J, Baker C, Bradbury A, Dhillon K, et al. Graduated compression stockings as adjuvant to pharmaco-thromboprophylaxis in elective surgical patients (GAPS study): randomised controlled trial. BMJ [Internet]. 2020 May 13 [cited 2020 Sep 25];369. Available from: https://www.bmj.com/content/369/bmj.m1309

12. CLOTS Trials Collaboration, Dennis M, Sandercock P a. G, Reid J, Graham C, Murray G, et al. Effectiveness of thigh-length graduated compression stockings to reduce the risk of deep vein thrombosis after stroke (CLOTS trial 1): a multicentre, randomised controlled trial. Lancet Lond Engl. 2009 Jun 6;373(9679):1958–65.

13. Hill J, Treasure T. Reducing the risk of venous thromboembolism (deep vein thrombosis and pulmonary embolism) in inpatients having surgery: summary of NICE guidance. BMJ. 2007 May 19;334(7602):1053–4.

14. Overview | Venous thromboembolism in over 16s: reducing the risk of hospital-acquired deep vein thrombosis or pulmonary embolism | Guidance | NICE [Internet]. NICE; [cited 2022 Mar 29]. Available from: https://www.nice.org.uk/guidance/ng89

15. Machin M, Younan HC, Smith S, Salim S, Davies AH, Shalhoub J. Systematic review on the benefit of graduated compression stockings in the prevention of venous thromboembolism in low-risk surgical patients. Phlebology. 2021 Apr;36(3):184–93.

16. Graduated compression stockings for prevention of deep vein thrombosis - Sachdeva, A - 2018 | Cochrane Library [Internet]. [cited 2022 Mar 29]. Available from: https://www.cochranelibrary.com/cdsr/doi/10.1002/14651858.CD001484.pub4/full

17. Department of Health. Risk assessment for Venous Thromboembolism (VTE) [Internet]. 2010 [cited 2021 May 11]. Available from: http://www.nice.org.uk/guidance/CG92

For peer review only - http://bmjopen.bmj.com/site/about/guidelines.xhtml

18. Measures of between-cluster variability in cluster randomized trials with binary outcomes - Thomson - 2009 - Statistics in Medicine - Wiley Online Library [Internet]. [cited 2022 Mar 29]. Available from: https://onlinelibrary.wiley.com/doi/10.1002/sim.3582

19. Unit Costs of Health and Social Care 2019 | PSSRU [Internet]. [cited 2022 Mar 29]. Available from: https://www.pssru.ac.uk/project-pages/unit-costs/unit-costs-2019/

20. Enden T, Resch S, White C, Wik HS, Kløw NE, Sandset PM. Cost-effectiveness of additional catheter-directed thrombolysis for deep vein thrombosis. J Thromb Haemost JTH. 2013 Jun;11(6):1032–42.

21. National Institute for Health and Care Excellence. Guide to the methods of technology appraisal 2013 ;94.

22. Husereau D, Drummond M, Augustovski F, de Bekker-Grob E, Briggs AH, Carswell C, et al. CHEERS 2022 ISPOR Good Research Practices Task Force. Consolidated Health Economic Evaluation Reporting Standards 2022 (CHEERS 2022) Statement: Updated Reporting Guidance for Health Economic Evaluations. Value Health. 2022 Jan;25(1):3-9.

For peer review only - http://bmjopen.bmj.com/site/about/guidelines.xhtml

Author contributions

The author contributions were as follows: Conception (AHD, JS, MM); Design of the work (AHD, JS, MM, SP, SW, BH, TE, MG, JN, DE, DW, CB, ZH, SS, LB, ASB, BG); Drafting the first version of the manuscript (SP); Critical review and revision of manuscript (AHD, JS, MM, SW, BH, TE, MG, JN, DE, DW, CB, ZH, SS, LB, ASB, BG).

Funding statement

This study is funded by the National Institute for Health Research (NIHR) Health Technology Assessment programme (NIHR133776). The views expressed are those of the author(s) and not necessarily those of the NIHR or the Department of Health and Social Care.

For peer review only

Competing interests

The authors have no competing interests to declare.

Current study status

The study is currently in the recruiting phase.

Trial sponsor

Imperial College London is the main sponsor for this study. Delegated responsibilities are assigned to the NHS Trusts/Health Boards taking part in this study.

Availability of data and materials

Data will be made available on reasonable request.

Abbreviations

AE	Adverse event

For peer review only - http://bmjopen.bmj.com/site/about/guidelines.xhtml

CI	Chief Investigator
DVT	Deep venous thrombosis
GCS	Graduated compression stockings
HAT	Hospital-acquired thrombosis
HRA	Health Research Authority
HTA	Health Technology Assessment
ICC	Intraclass correlation
ICER	Incremental Cost-Effectiveness Ratio
iDMC	Independent Data Monitoring Committee
LMWH	Low molecular weight heparin
NHS	National Health Service
NICE	National Institute for Health and Care Excellence
NIHR	National Institute for Health Research
NTT	Needed to treat
PE	Pulmonary embolism
PTS	Post-thrombotic syndrome
QALY	Quality adjusted life year
QoL	Quality of Life
RCT	Randomised controlled trial
REC	Research Ethics Committee
REDCap	Research Electronic Data Capture
SAE	Serious adverse event
TED	ThromboEmbolic Deterrent
TSC	Trial Steering Committee
VTE	Venous thromboembolism event

For peer review only

Figure legends

Figure 1. PETS Study Flow Chart

For peer review only - http://bmjopen.bmj.com/site/about/guidelines.xhtml

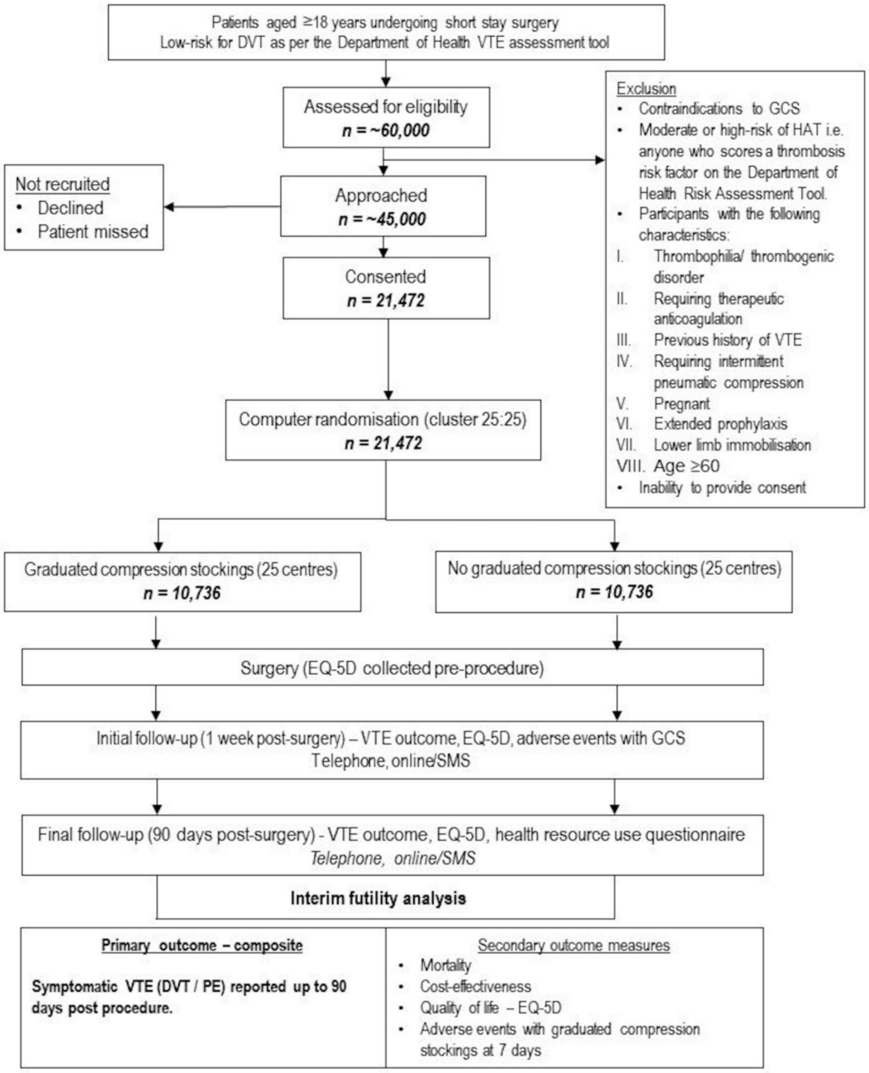

Figure 1. PETS Study Flow Chart

190x254mm (300 x 300 DPI)

SPIRIT

STANDARD PROTOCOL ITEMS: RECOMMENDATIONS FOR INTERVENTIONAL TRIALS

SPIRIT 2013 Checklist: Recommended items to address in a clinical trial protocol and related documents*

Section/item	Item No	Description
**Administrative information**		
Title	1	Descriptive title identifying the study design, population, interventions, and, if applicable, trial acronym
Trial registration	2a	Trial identifier and registry name. If not yet registered, name of intended registry
	2b	All items from the World Health Organization Trial Registration Data Set
Protocol version	3	Date and version identifier
Funding	4	Sources and types of financial, material, and other support
Roles and responsibilities	5a	Names, affiliations, and roles of protocol contributors
	5b	Name and contact information for the trial sponsor
	5c	Role of study sponsor and funders, if any, in study design; collection, management, analysis, and interpretation of data; writing of the report; and the decision to submit the report for publication, including whether they will have ultimate authority over any of these activities
	5d	Composition, roles, and responsibilities of the coordinating centre, steering committee, endpoint adjudication committee, data management team, and other individuals or groups overseeing the trial, if applicable (see Item 21a for data monitoring committee)
**Introduction**		
Background and rationale	6a	Description of research question and justification for undertaking the trial, including summary of relevant studies (published and unpublished) examining benefits and harms for each intervention
	6b	Explanation for choice of comparators
Objectives	7	Specific objectives or hypotheses
Trial design	8	Description of trial design including type of trial (eg, parallel group, crossover, factorial, single group), allocation ratio, and framework (eg, superiority, equivalence, noninferiority, exploratory)

For peer review only

Methods: Participants, interventions, and outcomes

Study setting	9	Description of study settings (eg, community clinic, academic hospital) and list of countries where data will be collected. Reference to where list of study sites can be obtained
Eligibility criteria	10	Inclusion and exclusion criteria for participants. If applicable, eligibility criteria for study centres and individuals who will perform the interventions (eg, surgeons, psychotherapists)
Interventions	11a	Interventions for each group with sufficient detail to allow replication, including how and when they will be administered
	11b	Criteria for discontinuing or modifying allocated interventions for a given trial participant (eg, drug dose change in response to harms, participant request, or improving/worsening disease)
	11c	Strategies to improve adherence to intervention protocols, and any procedures for monitoring adherence (eg, drug tablet return, laboratory tests)
	11d	Relevant concomitant care and interventions that are permitted or prohibited during the trial
Outcomes	12	Primary, secondary, and other outcomes, including the specific measurement variable (eg, systolic blood pressure), analysis metric (eg, change from baseline, final value, time to event), method of aggregation (eg, median, proportion), and time point for each outcome. Explanation of the clinical relevance of chosen efficacy and harm outcomes is strongly recommended
Participant timeline	13	Time schedule of enrolment, interventions (including any run-ins and washouts), assessments, and visits for participants. A schematic diagram is highly recommended (see Figure)
Sample size	14	Estimated number of participants needed to achieve study objectives and how it was determined, including clinical and statistical assumptions supporting any sample size calculations
Recruitment	15	Strategies for achieving adequate participant enrolment to reach target sample size

Methods: Assignment of interventions (for controlled trials)

Allocation:

Sequence generation	16a	Method of generating the allocation sequence (eg, computer-generated random numbers), and list of any factors for stratification. To reduce predictability of a random sequence, details of any planned restriction (eg, blocking) should be provided in a separate document that is unavailable to those who enrol participants or assign interventions

For peer review only - http://bmjopen.bmj.com/site/about/guidelines.xhtml

Allocation concealment mechanism	16b	Mechanism of implementing the allocation sequence (eg, central telephone; sequentially numbered, opaque, sealed envelopes), describing any steps to conceal the sequence until interventions are assigned
Implementation	16c	Who will generate the allocation sequence, who will enrol participants, and who will assign participants to interventions
Blinding (masking)	17a	Who will be blinded after assignment to interventions (eg, trial participants, care providers, outcome assessors, data analysts), and how
	17b	If blinded, circumstances under which unblinding is permissible, and procedure for revealing a participant's allocated intervention during the trial
**Methods: Data collection, management, and analysis**		
Data collection methods	18a	Plans for assessment and collection of outcome, baseline, and other trial data, including any related processes to promote data quality (eg, duplicate measurements, training of assessors) and a description of study instruments (eg, questionnaires, laboratory tests) along with their reliability and validity, if known. Reference to where data collection forms can be found, if not in the protocol
	18b	Plans to promote participant retention and complete follow-up, including list of any outcome data to be collected for participants who discontinue or deviate from intervention protocols
Data management	19	Plans for data entry, coding, security, and storage, including any related processes to promote data quality (eg, double data entry; range checks for data values). Reference to where details of data management procedures can be found, if not in the protocol
Statistical methods	20a	Statistical methods for analysing primary and secondary outcomes. Reference to where other details of the statistical analysis plan can be found, if not in the protocol
	20b	Methods for any additional analyses (eg, subgroup and adjusted analyses)
	20c	Definition of analysis population relating to protocol non-adherence (eg, as randomised analysis), and any statistical methods to handle missing data (eg, multiple imputation)
**Methods: Monitoring**		
Data monitoring	21a	Composition of data monitoring committee (DMC); summary of its role and reporting structure; statement of whether it is independent from the sponsor and competing interests; and reference to where further details about its charter can be found, if not in the protocol. Alternatively, an explanation of why a DMC is not needed

	21b	Description of any interim analyses and stopping guidelines, including who will have access to these interim results and make the final decision to terminate the trial
Harms	22	Plans for collecting, assessing, reporting, and managing solicited and spontaneously reported adverse events and other unintended effects of trial interventions or trial conduct
Auditing	23	Frequency and procedures for auditing trial conduct, if any, and whether the process will be independent from investigators and the sponsor
**Ethics and dissemination**		
Research ethics approval	24	Plans for seeking research ethics committee/institutional review board (REC/IRB) approval
Protocol amendments	25	Plans for communicating important protocol modifications (eg, changes to eligibility criteria, outcomes, analyses) to relevant parties (eg, investigators, REC/IRBs, trial participants, trial registries, journals, regulators)
Consent or assent	26a	Who will obtain informed consent or assent from potential trial participants or authorised surrogates, and how (see Item 32)
	26b	Additional consent provisions for collection and use of participant data and biological specimens in ancillary studies, if applicable
Confidentiality	27	How personal information about potential and enrolled participants will be collected, shared, and maintained in order to protect confidentiality before, during, and after the trial
Declaration of interests	28	Financial and other competing interests for principal investigators for the overall trial and each study site
Access to data	29	Statement of who will have access to the final trial dataset, and disclosure of contractual agreements that limit such access for investigators
Ancillary and post-trial care	30	Provisions, if any, for ancillary and post-trial care, and for compensation to those who suffer harm from trial participation
Dissemination policy	31a	Plans for investigators and sponsor to communicate trial results to participants, healthcare professionals, the public, and other relevant groups (eg, via publication, reporting in results databases, or other data sharing arrangements), including any publication restrictions
	31b	Authorship eligibility guidelines and any intended use of professional writers
	31c	Plans, if any, for granting public access to the full protocol, participant-level dataset, and statistical code

For peer review only

Appendices

Informed consent materials	32	Model consent form and other related documentation given to participants and authorised surrogates
Biological specimens	33	Plans for collection, laboratory evaluation, and storage of biological specimens for genetic or molecular analysis in the current trial and for future use in ancillary studies, if applicable

*It is strongly recommended that this checklist be read in conjunction with the SPIRIT 2013 Explanation & Elaboration for important clarification on the items. Amendments to the protocol should be tracked and dated. The SPIRIT checklist is copyrighted by the SPIRIT Group under the Creative Commons "Attribution-NonCommercial-NoDerivs 3.0 Unported" license.

For peer review only

BMJ Open

Examining the benefit of graduated compression stockings in the prevention of hospital-associated venous thromboembolism in low-risk surgical patients: a multicentre cluster randomised controlled trial (PETS Trial)

Journal:	*BMJ Open*
Manuscript ID	bmjopen-2022-069802.R1
Article Type:	Protocol
Date Submitted by the Author:	22-Nov-2022
Complete List of Authors:	Machin, Matthew; Imperial College London, Section of Vascular Surgery, Department of Surgery and Cancer, Charing Cross Hospital; Imperial College Healthcare NHS Trust, Imperial Vascular Unit, St Mary's Hospital<br>Peerbux, Sarrah; Imperial College London, Section of Vascular Surgery, Department of Surgery and Cancer, Charing Cross Hospital<br>Whittley, Sarah; Imperial College London, Section of Vascular Surgery, Department of Surgery and Cancer, Charing Cross Hospital<br>Hunt, Beverley; Guy's and St Thomas' NHS Foundation Trust, Thrombosis & Haemophilia Centre<br>Everington, Tamara; Hampshire Hospitals NHS Foundation Trust<br>Gohel, Manjit; Addenbrooke's Hospital, Department of Vascular Surgery<br>Norrie, John; Edinburgh Clinical Trials Unit, University of Edinburgh No. 9, Bioquarter, Usher Institute of Population Health Sciences and Informatics<br>Epstein, David; University of Granada, Faculty of Economic and Business Sciences<br>Warwick, David; University Hospital Southampton NHS Foundation Trust<br>Baker, Christopher; Imperial College Healthcare NHS Trust, Imperial Vascular Unit, St Mary's Hospital<br>Hamady, Zaed ; University Hospital Southampton NHS Foundation Trust, General surgery<br>Smith, Sasha; Imperial College London, Section of Vascular Surgery, Department of Surgery and Cancer, Charing Cross Hospital<br>Bolton, Layla; Imperial College Healthcare NHS Trust, Imperial Vascular Unit, St Mary's Hospital<br>Stephens-Boal, Annya; Imperial College London, Section of Vascular Surgery, Department of Surgery and Cancer, Charing Cross Hospital<br>Gray, Beverley; Imperial College London, Section of Vascular Surgery, Department of Surgery and Cancer, Charing Cross Hospital<br>Shalhoub, Joseph; Imperial College London, Section of Vascular Surgery, Department of Surgery and Cancer, Charing Cross Hospital; Imperial College Healthcare NHS Trust, Imperial Vascular Unit, St Mary's Hospital<br>Davies, AH; Imperial College London, Section of Vascular Surgery, Department of Surgery and Cancer, Charing Cross Hospital; Imperial College Healthcare NHS Trust, Imperial Vascular Unit, St Mary's Hospital
**Primary Subject Heading**:	Surgery

Secondary Subject Heading:	Anaesthesia, Cardiovascular medicine
Keywords:	Thromboembolism < CARDIOLOGY, SURGERY, VASCULAR MEDICINE

Title: Examining the benefit of graduated compression stockings in the prevention of hospital-associated venous thromboembolism in low-risk surgical patients: a multicentre cluster randomised controlled trial (PETS Trial)

Authors: Matthew Machin[1,2], Sarrah Peerbux[1], Sarah Whittley[1], Beverley J Hunt[3], Tamara Everington[4], Manjit Gohel[5], John Norrie[6], David Epstein[7], David Warwick[8], Christopher Baker[2], Zaed Hamady[8], Sasha Smith[1], Layla Bolton[2], Annya Stephens-Boal[1], Beverley Gray[1], Joseph Shalhoub[1,2], Alun H. Davies[1,2].

[1] Section of Vascular Surgery, Department of Surgery and Cancer, Imperial College London, Charing Cross Hospital, London W6 8RF, United Kingdom.

[2] Imperial Vascular Unit, Imperial College Healthcare NHS Trust, St Mary's Hospital, London W2 1NY, United Kingdom.

[3] Thrombosis & Haemophilia Centre, Guy's and St Thomas' NHS Foundation Trust, Westminster Bridge Road, London SE1 7EH, United Kingdom.

[4] Hampshire Hospitals NHS Foundation Trust, Aldermaston Road, Basingstoke, Hampshire, RG24 9NA, United Kingdom.

[5] Cambridge Vascular Unit, Addenbrookes Hospital, Cambridge University Hospitals NHS Foundation Trust, Hills Road, Cambridge CB2 0QQ, United Kingdom.

[6] Usher Institute of Population Health Sciences and Informatics, Edinburgh Clinical Trials Unit, University of Edinburgh, Level 2, NINE Edinburgh BioQuarter, 9 Little France Road, Edinburgh, EH16 4UX, United Kingdom

[7] Faculty of Economic and Business Sciences, University of Granada, Hospital Real, Avenida del Hospicio, S/N C.P. 18010, Granada, Spain.

[8] University Hospitals Southampton NHS Foundation Trust, Southampton General Hospital, Tremona Rd, Southampton SO16 6YD, United Kingdom.

For peer review only

Professor Alun Davies and Mr Joseph Shalhoub should be considered joint senior author.

Corresponding Author: Professor Alun H. Davies, Professor of Vascular Surgery, Section of Vascular Surgery, Department of Surgery and Cancer, Imperial College London, Charing Cross Hospital, London W6 8RF, United Kingdom. Email: a.h.davies@imperial.ac.uk.

Keywords: venous thromboembolism, graduated compression stockings, deep venous thrombosis, pulmonary embolism, cluster design.

For peer review only

ABSTRACT

Introduction: Hospital-acquired thrombosis (HAT) is defined as any venous thromboembolism (VTE) related event during a hospital admission or occurring up to 90 days post discharge, and is associated with significant morbidity, mortality and healthcare associated costs. Although surgery is an established risk factor for VTE, operations with a short hospital stay (<48 hours) and that permit early ambulation are associated with a low risk of VTE. Many patients undergoing short-stay surgical procedures and who are at low risk of VTE are treated with graduated compression stockings (GCS). However, evidence for the use of GCS in VTE prevention for this cohort is poor.

Methods and analysis: A multicentre, cluster randomised controlled trial which aims to determine whether GCS are superior in comparison to no GCS in the prevention of VTE for surgical patients undergoing short-stay procedures assessed to be at low risk of VTE. A total of 50 sites (21,472 participants) will be randomised to either intervention (GCS) or control (no GCS). Adult participants (18-59 years) who undergo short-stay surgical procedures and are assessed as low risk of VTE will be included in the study. Participants will provide consent to be contacted for follow-up at 7- and 90-days post-surgical procedure. The primary outcome is the rate of symptomatic VTE, i.e., DVT or PE during admission or within 90 days. Secondary outcomes include healthcare costs and changes in quality of life. The main analysis will be according to the intention-to-treat principle and will compare the rates of VTE at 90 days, measured at an individual level, using hierarchical (multilevel) logistic regression.

Ethics and dissemination: Ethical approval was granted by the Camden and Kings Cross Research

Ethics Committee (22/LO/0390). Findings will be published in a peer-reviewed journal and presented at national and international conferences.

Registration details: ISRCTN13908683.

ARTICLE SUMMARY

Strengths and Limitations of the study

- This is the first randomised controlled trial evaluating the role of GCS in the prevention of hospital-associated VTE in short-stay surgical patients who are assessed as low risk of developing hospital-associated VTE
- The primary outcome is clinically meaningful and objective in nature
- The cost-effectiveness analysis will assess the economic implications of providing GCS for the prevention of VTE in low risk, short-stay surgical patients
- Given this is a cluster design, it will be important to demonstrate that the populations are balanced in terms of confounding factors i.e., that the clustering has been successful

Word count: 3,444

For peer review only - http://bmjopen.bmj.com/site/about/guidelines.xhtml

INTRODUCTION

Hospital-associated thrombosis (HAT) is defined as any venous thromboembolism (VTE) related event occurring during admission and/or within 90 days of hospital admission (1) and is a term that encompasses both deep vein thrombosis (DVT) and pulmonary embolism (PE). HAT accounts for significant morbidity and mortality with 57.1 VTE-related deaths per 100,000 hospital admissions reported in the year 2018-2019 within the UK National Health Service (NHS) (1). The probability of untreated moderate-risk surgical inpatients developing HAT was previously estimated to be as high as 15%, this reducing to 4.1% with pharmacological and mechanical prophylaxis (2).

Previously, UK annual VTE-related mortality was estimated to be 32,000 fatalities if thromboprophylaxis was not used, with associated costs of £640 million per annum (3). Surgery is an established risk factor for VTE (4). It is estimated that the risk of VTE for patients undergoing most general surgical, urological or open gynaecological procedures without thromboprophylaxis is 10-40% (5). Furthermore, for those undergoing hip or knee arthroplasty, this risk increases to 40-80% (5). Specific surgical factors such as abdominal and pelvic procedures, operations for cancer, and procedures with a greater duration are associated with a greater risk of VTE (6,7). Conversely, operations with short anaesthetic and procedure times, that can be performed within a <48-hour hospital stay, and that permit early ambulation are associated with a low risk of VTE (8).

Mechanical thromboprophylaxis in the form of graduated compression stockings (GCS), also known by a brand name ThromboEmbolic Deterrent (TED) (5,9,10), is often used. GCS apply a level of graduated pressure at rest, i.e., not whilst ambulant; they are designed for immobile patients at risk of VTE. However, it has recently been shown that GCS provide no added benefit in the reduction of HAT for moderate and high VTE risk surgical inpatients receiving prophylactic dose of low molecular weight heparin (LMWH) (11). Additionally, the CLOTS 1 trial, which randomised acute stroke patients to either standard care alone or the combination of GCS and standard care, reported no difference in the rate of DVT (12). The findings of these and other studies have cast doubt on the use of GCS in prevention of VTE. Importantly, the patients in the aforementioned trials were at a higher risk from the distinct low VTE risk group.

Limited evidence is available on the rate of HAT in low-risk surgical patients. In the UK, National Institute for Health and Care Excellence (NICE) guidelines for the prevention of VTE published in 2007

For peer review only - http://bmjopen.bmj.com/site/about/guidelines.xhtml

previously recommended that all surgical patients should receive GCS to reduce the risk of VTE, irrespective of the absence of thrombosis risk factors (13). The subsequent updated NICE guidelines did not include this blanket recommendation, but instead provided specific recommendations for differing procedure types. The most contemporary NICE guidelines, published in 2018, recommend that all patients undergoing abdominal, thoracic, spinal, bariatric, head and neck, and elective joint surgery should be treated with GCS, and to consider treatment with GCS for all those undergoing cardiac, vascular and ear, nose and throat surgery (14). The interpretation of these recommendations has meant that patients undergoing short-stay procedures, who are able to ambulate early and have no other thrombosis risk factors, are still treated with GCS. Hence, it is now common practice for patients undergoing day case procedures, even those that have no other assessed risk factors, to be prescribed GCS in the absence of contraindications.

The evidence to support GCS for short-stay ambulant patients is poor. A systematic review (15) examining the use of GCS in comparison to no prophylaxis in low VTE risk short stay surgical patients confirmed that there have been no randomised controlled trials (RCTs) to support this practice. Furthermore, a meta-analysis in the 2018 Cochrane Review for patients at moderate and high-risk of VTE revealed a significantly lower odds ratio of DVT in those treated with GCS in comparison to those that were not (16). However, none of the included 18 surgical RCTs identified in the review consisted of low-risk procedures, i.e. ambulatory day case procedures.

Moreover, the UK National Institute for Health Research (NIHR) Health Technology Assessment (HTA)-funded GAPS trial, assessing the use of GCS in addition to LMWH for the prevention of VTE in moderate and high-risk (i.e. not low risk) elective surgical patients, demonstrating that LMWH alone was non-inferior to dual thromboprophylaxis with LMWH and GCS, has further drawn the role of GCS in the prevention of VTE in surgery into question (11). This is on the background of other RCTs, such as the CLOTS 1 trial which randomised acute stroke patients to either standard care or the combination of GCS and standard care and reported no difference in the rate of DVT (12). While the clinical community are adopting the GAPS trial findings into clinical practice, they cannot safely be extrapolated to this large cohort of low VTE risk patients who don't also receive LMWH.

For peer review only

For peer review only - http://bmjopen.bmj.com/site/about/guidelines.xhtml

Hence, due to this paucity of evidence to support the use of GCS in the prevention of VTE for low VTE risk surgical patients, an adequately powered trial is required to provide evidence in relation to this practice.

Objectives

The primary objective is to evaluate the potential benefit of GCS in the prevention of HAT in patients undergoing short-stay surgical procedures, assessed as being at low risk for VTE. Secondary objectives include comparisons of quality of life (QoL) at 7- and 90-days, mortality at 90-days, and a cost-effectiveness analysis.

METHODS AND ANALYSIS

Trial design

This is a prospective, multicentre, cluster randomised controlled trial with a follow-up of three months. The primary outcome is assessed blindly.

Study Setting

Eligible participants will be recruited from at least 50 NHS Trusts/Health Boards sites in the United Kingdom. A complete list of actively recruiting study sites can be obtained from https://clinicaltrials.gov/ct2/show/NCT05347550.

Eligibility Criteria

Inclusion criteria are: adult patients aged 18-59 years of age (as age 60 scores 1 point on risk assessment) scheduled to undergo a surgical procedure with a hospital stay <48 hours who are assessed as being at low risk of developing VTE as per the Department of Health risk assessment tool (i.e. no assessed thrombosis risk factors / scoring 0) (17). Exclusion criteria include individuals: with a contraindication to GCS; assessed as being at moderate or high-risk of VTE as per the Department of Health risk assessment tool; requiring therapeutic anticoagulation; with thrombophilia or thrombotic disorders; with a previous history of VTE; requiring intermittent pneumatic compression therapy beyond theatre and recovery; requiring extended thromboprophylaxis beyond discharge; female of childbearing

For peer review only - http://bmjopen.bmj.com/site/about/guidelines.xhtml

age who have a positive pregnancy test; those with lower limb immobilisation; those unable to provide informed consent.

Interventions

Centres randomised to the intervention arm, which is the current standard of care, will consist of participants receiving GCS. Clinical staff (e.g., theatre support workers) will issue stockings to all patients who are scheduled to undergo short-stay surgery. Participants will be instructed to wear their stockings just before undergoing the surgical procedure and to remove the stockings as soon as they are ambulant after the procedure.

In those centres randomised to the control arm, participants will not receive GCS.

Figure 1 is a diagram displaying the participant flow in the trial.

Primary outcome

The primary outcome is the rate of symptomatic VTE during admission and/or within 90 days after discharge for surgical patients undergoing short-stay procedures assessed as being at low risk of VTE, in those treated with or without GCS.

Secondary outcomes

The secondary outcomes include:

- Mortality rates in each group
- Adverse events related to the GCS (assessed at 7-days, in those enrolled in the intervention clusters only)
- QoL score at 7- and 90-days post-surgical procedure assessed using the EQ-5D
- Resource use and costs of healthcare, and days off work and normal activities, up to 90 days

Sample size and study duration

Good quality data for the incidence of VTE in low VTE risk surgical patients not receiving thromboprophylaxis is lacking. Data from the recently published GAPS RCT on moderate and high risk patients reported 29/1858 (1.6%) VTE events at 90 days (11). The VTE rate for short stay procedures

For peer review only

in individuals formally assessed as being at low risk of VTE would be expected to be lower than this, however, as these short stay patients are not receiving a thromboprophylactic dose of LMWH, we have conservatively assumed an overall control (no stockings nor drugs) VTE rate of 1.0%.

The sample size calculation is for a superiority comparison based on symptomatic VTE (DVT or PE) at 90 days at 90% power with a significance level of 5%, assuming 1.0% in the no stocking (control) and 0.5% in the stocking (active) groups.

Assuming an intraclass correlation coefficient (ICC) on the VTE at 90 days outcome of 0.001 (consistent with the control outcome rate varying uniformly between 0.5% and 1.5%) (18) and a coefficient of variation of site size of 0.25 (consistent with sites varying according to a Normal distribution mean=350 and SD=88 i.e. 95% of the sites would be between 174 and 526 participants), with 25 sites randomised to active and 25 sites to control, the study would need to recruit 17,500 patients. Allowing for 15% loss to follow, but no sites lost to follow up, and adjusting for the group sequential design with one formal interim analysis at 50% with 90-day follow up, 21,472 participants are required. We do not believe there will be any meaningful crossover. A check on the sample size assumptions will be incorporated when there are 10 sites across both arms that have recruited at least 100 patients each (checking on variability in the control outcome for ICC and checking zero crossover). At this stage, as well as confirming the overall maximum sample size, the schedule for a single efficacy or futility analysis at around 50% of the target recruitment with mature 90-day follow-up will be included.

For peer review only

Interim analysis

When 50% mature primary outcome data is available, a formal interim analysis will be performed with the possibility of stopping early for futility (no prospect of a clinically meaningful treatment effect).

Recruitment and randomisation

This is a cluster study, hence randomisation will be conducted at recruitment centre level rather than on an individual participant level. Each site will confirm capacity and capability with their local R&D team for participation in the trial prior to randomisation. Sites will be randomised by the Edinburgh Clinical Trials Unit by simple randomisation (1:1) via the Research Electronic Data Capture (REDCap) database, and will be adjusted for based on region, size and VTE compliance within the Trust. The Trial Manager will be informed of the treatment allocation via email, and the Trust will be informed via a

For peer review only - http://bmjopen.bmj.com/site/about/guidelines.xhtml

Treatment Allocation Letter. Sites and the study team will undertake local engagement to identify appropriate and willing specialties which form the basic centre unit. Recruiting centres will be randomised/allocated to either the intervention arm (where participants who undergo short-stay surgery will receive GCS to wear during their short-stay procedure) or the control arm (where no GCS will be provided to those undergoing short-stay surgery).

Due to the nature of this cluster design, patients are only required to consent to be contacted for follow-up (at 7- and 90-days post procedure).

Patients from a variety of surgical specialties will be included in this pragmatic trial. Adults who are scheduled to undergo short-stay surgery will be pre-screened by a member of the direct care team and invited to speak to a member of the research team. Permission from the patient will be granted by the direct care team before any information is passed or approach made by the research team.

Recruitment will commence on 01/10/2022 for 30-months. The study will close on 31/12/2025.

Blinding

The primary outcome is assessed blindly. Follow-up data will be collected centrally by blinded assessors at the coordinating centre (Imperial College London).

Follow-up periods

Participants will be followed-up by at 7- and 90-days post-surgical procedure. Follow-up data will be collected via telephone or online questionnaire (the link to the questionnaire will be sent via email or SMS).

At 7-days post-surgical procedure, data on self-reported VTE outcome will be collected and the EQ-5D will be administered. For those participants who were enrolled in the intervention cluster only (i.e., sites which were randomised to issue GCS to short-stay surgical patients), information on adverse events related to GCS will also be collected.

At 90-days post-surgical procedure, data on self-reported VTE outcome will be collected, together with the EQ-5D and information on the healthcare resource use.

Data collection and confidentiality

For peer review only - http://bmjopen.bmj.com/site/about/guidelines.xhtml

Participant data will be collected and entered onto the web-based database REDCap by the local research team. Data will be filed for ten years as per local policy and then deleted. Data will be monitored by the Trial Manager for quality and completeness, and missing data will be requested from the participating sites, as per the data monitoring plan. The Trial Manager will only have access to pseudonymised data on REDCap.

Statistical analysis

All statistical analyses will be governed by a comprehensive Statistical Analysis Plan, authored by the study statisticians and agreed by the independent Trial Steering Committee (TSC).

The main analysis will be according to the intention-to-treat principle and will compare the rates of VTE at 90 days, measured at an individual level, using hierarchical (multilevel) logistic regression, adjusting for any other pre-specified strongly prognostic individual baseline covariates and site-level baseline covariates, with site itself as a random effect. This will be performed independently by the Edinburgh Clinical Trials Unit who will have sole access to the final REDCap dataset. The findings will be assessed for robustness against any missing data, first using multiple imputation assuming this data is missing at random and, if appropriate and the data permits, further sensitivity analyses will be attempted under any plausible missing data mechanisms not missing at random. Secondary outcomes will be analysed in a similar fashion with generalised linear models appropriate to the distribution of the outcome. Safety data will be summarised descriptively.

Internal pilot

There will be an internal pilot of feasibility at the end of 6 months of recruitment (beginning of month 7 to end of month 12 of the overall study timeline). We will start recruiting the (minimum) 50 sites at four sites per month. The 100% recruitment rate to meet the sample size is 20 participants per centre month (accounting for the staggered site set-up). This equates to a target of 1440 participants at the end of the 6-month internal pilot. A stringent criterion of <5% failure to receive allocated intervention is set as a marker of crossover between trial arms. Regarding follow-up, a marker of <15% lost to follow-up would be indicative of green progression. The amber criteria is set at 75 – 99% of recruitment. This equates to a minimum of 1,080 participants across the 6-month internal pilot period and an associated mean recruitment rate of 15 participants per centre-month. We have set an amber criterion for site set-

For peer review only - http://bmjopen.bmj.com/site/about/guidelines.xhtml

up independent of total recruitment number, hence, even if the total participant number is at 100%, if the number of sites set-up is <24 then this would stimulate a review of the site set-up strategy. Any amber criteria would prompt a review of the trial methodology and recruitment strategy. The red criteria, i.e., consideration of stopping the trial early, is set at <75% recruitment and is equivalent to achieving fewer than 1,080 participants across the 6-month internal pilot period. An independent threshold for site set-up has been set at <18 sites. The TSC will meet at the end of month 13 (allowing 1 month of processing time after the 6 months of recruitment) with blinded estimation of the rate of VTE for each arm. This will confirm the sample size or lead to a sample size re-estimation.

Cost-effectiveness analysis

Two health economic analyses will be conducted. The main analyses will be performed from the perspective of the NHS and Personal Social Services, with secondary analyses from a societal perspective.

A within-trial analysis will compare GCS to no thromboprophylaxis over the 90 days of the study. Resource use items associated with treatments in hospital and community care will be collected using case notes and self-completed patient resource use diaries over the 90-day follow-up, and costed using manufacturers' list prices, previous literature, and national reference costs. Days off work and normal activities and other patient-related costs will be collected for a secondary analysis. EQ-5D will be collected at baseline and follow up, analysed using the NICE approved tariff. Appropriate methods will be used to handle missing data and any relevant subgroups in line with the Statistical Analysis Plan.

If there are clinically relevant and measurable differences in VTE or quality of life between the study arms at 90 days, a Markov (state–transition) decision model will be constructed to compare the ICER up to 2 years for GCS versus no GCS. The time horizon of the model will be 2 years allowing extrapolation of sequela of VTE events (such as post-thrombotic syndrome [PTS]) over the longer term to quantify the impact of VTE on patient health (quality adjusted life years [QALYs]) and resource use. A preliminary model has been constructed based on published literature to identify the key variables that would need to be collected during the clinical study, and to estimate the number needed to treat (NNT) to avoid 1 VTE, above which GCS would not be considered cost-effective at NICE thresholds. The 2-year time point was chosen as we know from previous research that the incidence of PTS after acute DVT levels out after the first year.

For peer review only - http://bmjopen.bmj.com/site/about/guidelines.xhtml

This preliminary model conservatively assumes 30% of patients with VTE develop PTS, with 3% of those patients having severe PTS. The cost of purchasing and applying GCS stockings is approximately £22.46 (19). The model assumes the cost of treatment of VTE, non-severe and severe PTS, are £451, £872 and £1547, respectively, and estimates of the utility decrement associated with symptomatic VTE and PTS which are 0.8628, 0.7745 and 0.6752, respectively (20). Using a 2-year time horizon, the ICER of GCS versus no prophylaxis would be £20,603 per QALY if the NNT were 200 participants. Hence, for GCS to be cost-effective at a NICE willingness to pay threshold, the NNT would need to be below 200.

The health economic analyses will be conducted and reported according to NICE reference case and CHEERS guidelines (21,22), including sensitivity analyses and probabilistic sensitivity analyses. The results will be presented as estimates of mean incremental costs, effects, and incremental cost per QALY.

Data monitoring, safety and quality control

An independent TSC and independent Data Monitoring Committee (iDMC) have been appointed. The main role of the TSC will be to provide overall supervision of the trial and ensure that it is being conducted in accordance with the principles of Good Clinical Practice and the relevant regulations. The main role of the iDMC will be to safeguard the interests of trial participants and to monitor the main outcome measures including safety and efficacy. The Clinical Trial Manager, together with the Trial Management Group, will oversee trial progress.

All treatment-related adverse events (AEs; related to the GCS only) will be collected centrally at 7-days post-procedure, as will all serious adverse events (SAEs). The chief investigator will be notified of all SAEs within 24 hours. All SAEs will be reported to the research ethics committee (REC) and sponsor if, in the opinion of the CI, the event was related to the intervention. These analyses will be descriptive, with any p-values calculated to be interpreted descriptively.

Patient and public involvement

During the grant application stage, online surveys were launched to learn public views on the PETS study. These surveys helped to inform important aspects of the trial, including the number of visits and questionnaires used in the study. Two patient advisors, both with lived experience of VTE, have been involved in the design of the study and review of patient facing materials. Both were grant co-applicants,

For peer review only

For peer review only - http://bmjopen.bmj.com/site/about/guidelines.xhtml

have agreed to sit on the Trial Steering Committee, facilitate patient advisory groups, will assist with the dissemination of results, and are co-authors of this manuscript.

ETHICS AND DISSEMINATION

Ethical approval was granted by the Camden and Kings Cross Research Ethics Committee (22/LO/0390). Amendments to the protocol will be updated on the ISRCTN record. All amendments to the protocol will be submitted to the sponsor for review before applying for approval from the REC and the Health Research Authority (HRA). Protocol amendments will be circulated by email to investigators and study nurses. Standard informed consent will be obtained by research nurses and investigators, with freedom to withdraw at any time. The findings from this study will be published in peer-reviewed journals, presented at national and international conferences and disseminated to participants (via emails and letters at the end of the study).

For peer review only - http://bmjopen.bmj.com/site/about/guidelines.xhtml

REFERENCES

1. 5.1 Deaths from venous thromboembolism (VTE) related events within 90 days post discharge from hospital [Internet]. NHS Digital. [cited 2022 Mar 29]. Available from: https://digital.nhs.uk/data-and-information/publications/statistical/nhs-outcomes-framework/march-2022/domain-5---treating-and-caring-for-people-in-a-safe-environment-and-protecting-them-from-avoidable-harm-nof/5.1-deaths-from-venous-thromboembolism-vte-related-events-within-90-days-post-discharge-from-hospital

2. Cohen AT, Agnelli G, Anderson FA, Arcelus JI, Bergqvist D, Brecht JG, et al. Venous thromboembolism (VTE) in Europe. The number of VTE events and associated morbidity and mortality. Thromb Haemost. 2007 Oct;98(4):756–64.

3. The Prevention of Venous Thromboembolism in Hospitalised Patients Second Report of Session 2004-05.

4. Anderson FA, Spencer FA. Risk factors for venous thromboembolism. Circulation. 2003 Jun 17;107(23 Suppl 1):I9-16.

5. Geerts WH, Bergqvist D, Pineo GF, Heit JA, Samama CM, Lassen MR, et al. Prevention of venous thromboembolism: American College of Chest Physicians Evidence-Based Clinical Practice Guidelines (8th Edition). Chest. 2008 Jun;133(6 Suppl):381S-453S.

6. Gould MK, Garcia DA, Wren SM, Karanicolas PJ, Arcelus JI, Heit JA, et al. Prevention of VTE in nonorthopedic surgical patients: Antithrombotic Therapy and Prevention of Thrombosis, 9th ed: American College of Chest Physicians Evidence-Based Clinical Practice Guidelines. Chest. 2012 Feb;141(2 Suppl):e227S-e277S.

7. Sweetland S, Green J, Liu B, Berrington de González A, Canonico M, Reeves G, et al. Duration and magnitude of the postoperative risk of venous thromboembolism in middle aged women: prospective cohort study. BMJ. 2009 Dec 3;339:b4583.

8. Hair A, Duffy K, McLean J, Taylor S, Smith H, Walker A, et al. Groin hernia repair in Scotland. Br J Surg. 2000 Dec;87(12):1722–6.

For peer review only - http://bmjopen.bmj.com/site/about/guidelines.xhtml

9. Overview | Venous thromboembolic diseases: diagnosis, management and thrombophilia testing | Guidance | NICE [Internet]. NICE; [cited 2022 Mar 29]. Available from: https://www.nice.org.uk/guidance/ng158

10. Compression stockings | Health topics A to Z | CKS | NICE [Internet]. [cited 2022 Mar 29]. Available from: https://cks.nice.org.uk/topics/compression-stockings/

11. Shalhoub J, Lawton R, Hudson J, Baker C, Bradbury A, Dhillon K, et al. Graduated compression stockings as adjuvant to pharmaco-thromboprophylaxis in elective surgical patients (GAPS study): randomised controlled trial. BMJ [Internet]. 2020 May 13 [cited 2020 Sep 25];369. Available from: https://www.bmj.com/content/369/bmj.m1309

12. CLOTS Trials Collaboration, Dennis M, Sandercock P a. G, Reid J, Graham C, Murray G, et al. Effectiveness of thigh-length graduated compression stockings to reduce the risk of deep vein thrombosis after stroke (CLOTS trial 1): a multicentre, randomised controlled trial. Lancet Lond Engl. 2009 Jun 6;373(9679):1958–65.

13. Hill J, Treasure T. Reducing the risk of venous thromboembolism (deep vein thrombosis and pulmonary embolism) in inpatients having surgery: summary of NICE guidance. BMJ. 2007 May 19;334(7602):1053–4.

14. Overview | Venous thromboembolism in over 16s: reducing the risk of hospital-acquired deep vein thrombosis or pulmonary embolism | Guidance | NICE [Internet]. NICE; [cited 2022 Mar 29]. Available from: https://www.nice.org.uk/guidance/ng89

15. Machin M, Younan HC, Smith S, Salim S, Davies AH, Shalhoub J. Systematic review on the benefit of graduated compression stockings in the prevention of venous thromboembolism in low-risk surgical patients. Phlebology. 2021 Apr;36(3):184–93.

16. Graduated compression stockings for prevention of deep vein thrombosis - Sachdeva, A - 2018 | Cochrane Library [Internet]. [cited 2022 Mar 29]. Available from: https://www.cochranelibrary.com/cdsr/doi/10.1002/14651858.CD001484.pub4/full

17. Department of Health. Risk assessment for Venous Thromboembolism (VTE) [Internet]. 2010 [cited 2021 May 11]. Available from: http://www.nice.org.uk/guidance/CG92

For peer review only

18. Measures of between-cluster variability in cluster randomized trials with binary outcomes - Thomson - 2009 - Statistics in Medicine - Wiley Online Library [Internet]. [cited 2022 Mar 29]. Available from: https://onlinelibrary.wiley.com/doi/10.1002/sim.3582

19. Unit Costs of Health and Social Care 2019 | PSSRU [Internet]. [cited 2022 Mar 29]. Available from: https://www.pssru.ac.uk/project-pages/unit-costs/unit-costs-2019/

20. Enden T, Resch S, White C, Wik HS, Kløw NE, Sandset PM. Cost-effectiveness of additional catheter-directed thrombolysis for deep vein thrombosis. J Thromb Haemost JTH. 2013 Jun;11(6):1032–42.

21. National Institute for Health and Care Excellence. Guide to the methods of technology appraisal 2013 ;94.

22. Husereau D, Drummond M, Augustovski F, de Bekker-Grob E, Briggs AH, Carswell C, et al. CHEERS 2022 ISPOR Good Research Practices Task Force. Consolidated Health Economic Evaluation Reporting Standards 2022 (CHEERS 2022) Statement: Updated Reporting Guidance for Health Economic Evaluations. Value Health. 2022 Jan;25(1):3-9.

For peer review only - http://bmjopen.bmj.com/site/about/guidelines.xhtml

Author contributions

The author contributions were as follows: Conception (AHD, JS, MM); Design of the work (AHD, JS, MM, SP, SW, BH, TE, MG, JN, DE, DW, CB, ZH, SS, LB, ASB, BG); Drafting the first version of the manuscript (SP); Critical review and revision of manuscript (AHD, JS, MM, SW, BH, TE, MG, JN, DE, DW, CB, ZH, SS, LB, ASB, BG).

Funding statement

This study is funded by the National Institute for Health Research (NIHR) Health Technology Assessment programme (NIHR133776). The views expressed are those of the author(s) and not necessarily those of the NIHR or the Department of Health and Social Care.

Competing interests

The authors have no competing interests to declare.

Current study status

The study is currently in the recruiting phase.

Trial sponsor

Imperial College London is the main sponsor for this study. Delegated responsibilities are assigned to the NHS Trusts/Health Boards taking part in this study.

Availability of data and materials

Data will be made available on reasonable request.

Current protocol version and date

Protocol V2.0 (dated 18/07/2022)

For peer review only - http://bmjopen.bmj.com/site/about/guidelines.xhtml

Abbreviations

AE	Adverse event
CI	Chief Investigator
DVT	Deep venous thrombosis
GCS	Graduated compression stockings
HAT	Hospital-acquired thrombosis
HRA	Health Research Authority
HTA	Health Technology Assessment
ICC	Intraclass correlation
ICER	Incremental Cost-Effectiveness Ratio
iDMC	Independent Data Monitoring Committee
LMWH	Low molecular weight heparin
NHS	National Health Service
NICE	National Institute for Health and Care Excellence
NIHR	National Institute for Health Research
NTT	Needed to treat
PE	Pulmonary embolism
PTS	Post-thrombotic syndrome
QALY	Quality adjusted life year
QoL	Quality of Life
RCT	Randomised controlled trial
REC	Research Ethics Committee
REDCap	Research Electronic Data Capture
SAE	Serious adverse event
TED	ThromboEmbolic Deterrent
TSC	Trial Steering Committee
VTE	Venous thromboembolism event

For peer review only

For peer review only - http://bmjopen.bmj.com/site/about/guidelines.xhtml

497 **Figure legends**

498 **Figure 1.** PETS Study Flow Chart

For peer review only

For peer review only - http://bmjopen.bmj.com/site/about/guidelines.xhtml

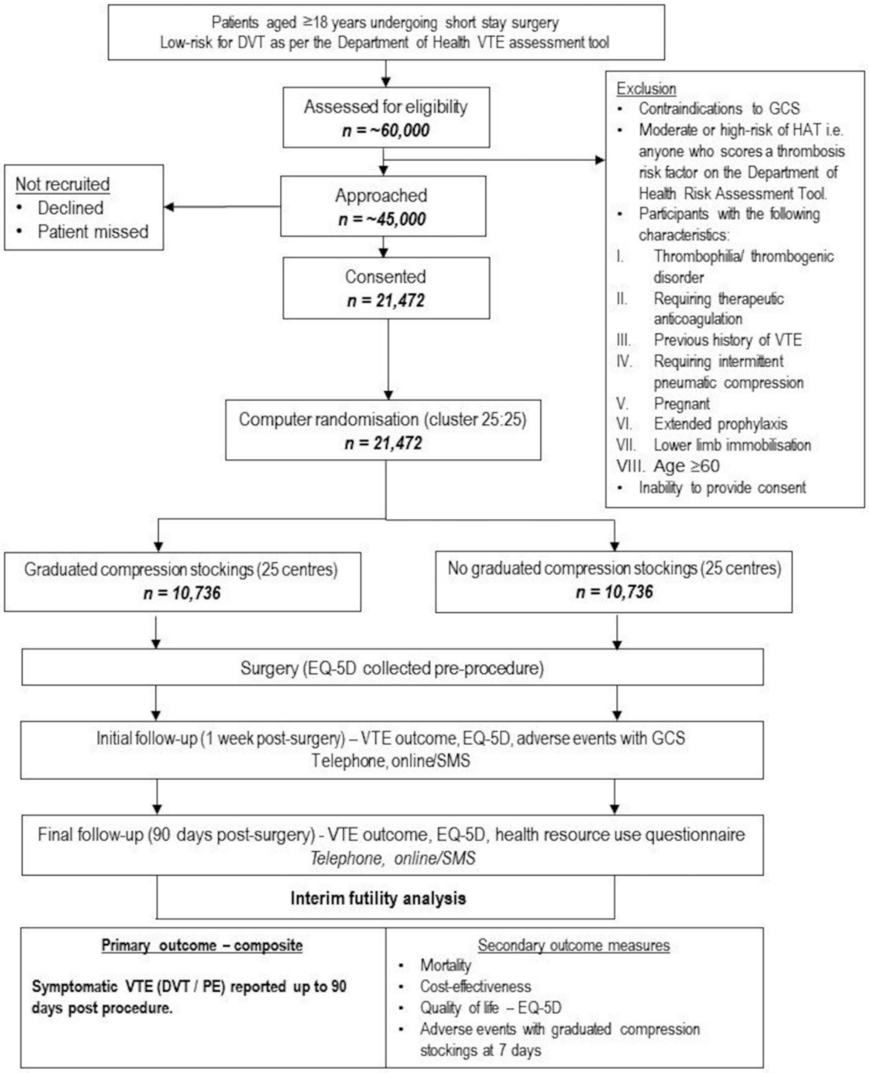

Figure 1. PETS Study Flow Chart

190x254mm (300 x 300 DPI)

SPIRIT

STANDARD PROTOCOL ITEMS: RECOMMENDATIONS FOR INTERVENTIONAL TRIALS

SPIRIT 2013 Checklist: Recommended items to address in a clinical trial protocol and related documents*

Section/item	Item No	Description
**Administrative information**		
Title	1	Descriptive title identifying the study design, population, interventions, and, if applicable, trial acronym
Trial registration	2a	Trial identifier and registry name. If not yet registered, name of intended registry
	2b	All items from the World Health Organization Trial Registration Data Set
Protocol version	3	Date and version identifier
Funding	4	Sources and types of financial, material, and other support
Roles and responsibilities	5a	Names, affiliations, and roles of protocol contributors
	5b	Name and contact information for the trial sponsor
	5c	Role of study sponsor and funders, if any, in study design; collection, management, analysis, and interpretation of data; writing of the report; and the decision to submit the report for publication, including whether they will have ultimate authority over any of these activities
	5d	Composition, roles, and responsibilities of the coordinating centre, steering committee, endpoint adjudication committee, data management team, and other individuals or groups overseeing the trial, if applicable (see Item 21a for data monitoring committee)
**Introduction**		
Background and rationale	6a	Description of research question and justification for undertaking the trial, including summary of relevant studies (published and unpublished) examining benefits and harms for each intervention
	6b	Explanation for choice of comparators
Objectives	7	Specific objectives or hypotheses
Trial design	8	Description of trial design including type of trial (eg, parallel group, crossover, factorial, single group), allocation ratio, and framework (eg, superiority, equivalence, noninferiority, exploratory)

For peer review only - http://bmjopen.bmj.com/site/about/guidelines.xhtml

Methods: Participants, interventions, and outcomes

Study setting	9	Description of study settings (eg, community clinic, academic hospital) and list of countries where data will be collected. Reference to where list of study sites can be obtained
Eligibility criteria	10	Inclusion and exclusion criteria for participants. If applicable, eligibility criteria for study centres and individuals who will perform the interventions (eg, surgeons, psychotherapists)
Interventions	11a	Interventions for each group with sufficient detail to allow replication, including how and when they will be administered
	11b	Criteria for discontinuing or modifying allocated interventions for a given trial participant (eg, drug dose change in response to harms, participant request, or improving/worsening disease)
	11c	Strategies to improve adherence to intervention protocols, and any procedures for monitoring adherence (eg, drug tablet return, laboratory tests)
	11d	Relevant concomitant care and interventions that are permitted or prohibited during the trial
Outcomes	12	Primary, secondary, and other outcomes, including the specific measurement variable (eg, systolic blood pressure), analysis metric (eg, change from baseline, final value, time to event), method of aggregation (eg, median, proportion), and time point for each outcome. Explanation of the clinical relevance of chosen efficacy and harm outcomes is strongly recommended
Participant timeline	13	Time schedule of enrolment, interventions (including any run-ins and washouts), assessments, and visits for participants. A schematic diagram is highly recommended (see Figure)
Sample size	14	Estimated number of participants needed to achieve study objectives and how it was determined, including clinical and statistical assumptions supporting any sample size calculations
Recruitment	15	Strategies for achieving adequate participant enrolment to reach target sample size

Methods: Assignment of interventions (for controlled trials)

Allocation:

Sequence generation	16a	Method of generating the allocation sequence (eg, computer-generated random numbers), and list of any factors for stratification. To reduce predictability of a random sequence, details of any planned restriction (eg, blocking) should be provided in a separate document that is unavailable to those who enrol participants or assign interventions

For peer review only - http://bmjopen.bmj.com/site/about/guidelines.xhtml

Allocation concealment mechanism	16b	Mechanism of implementing the allocation sequence (eg, central telephone; sequentially numbered, opaque, sealed envelopes), describing any steps to conceal the sequence until interventions are assigned
Implementation	16c	Who will generate the allocation sequence, who will enrol participants, and who will assign participants to interventions
Blinding (masking)	17a	Who will be blinded after assignment to interventions (eg, trial participants, care providers, outcome assessors, data analysts), and how
	17b	If blinded, circumstances under which unblinding is permissible, and procedure for revealing a participant's allocated intervention during the trial
**Methods: Data collection, management, and analysis**		
Data collection methods	18a	Plans for assessment and collection of outcome, baseline, and other trial data, including any related processes to promote data quality (eg, duplicate measurements, training of assessors) and a description of study instruments (eg, questionnaires, laboratory tests) along with their reliability and validity, if known. Reference to where data collection forms can be found, if not in the protocol
	18b	Plans to promote participant retention and complete follow-up, including list of any outcome data to be collected for participants who discontinue or deviate from intervention protocols
Data management	19	Plans for data entry, coding, security, and storage, including any related processes to promote data quality (eg, double data entry; range checks for data values). Reference to where details of data management procedures can be found, if not in the protocol
Statistical methods	20a	Statistical methods for analysing primary and secondary outcomes. Reference to where other details of the statistical analysis plan can be found, if not in the protocol
	20b	Methods for any additional analyses (eg, subgroup and adjusted analyses)
	20c	Definition of analysis population relating to protocol non-adherence (eg, as randomised analysis), and any statistical methods to handle missing data (eg, multiple imputation)
**Methods: Monitoring**		
Data monitoring	21a	Composition of data monitoring committee (DMC); summary of its role and reporting structure; statement of whether it is independent from the sponsor and competing interests; and reference to where further details about its charter can be found, if not in the protocol. Alternatively, an explanation of why a DMC is not needed

	21b	Description of any interim analyses and stopping guidelines, including who will have access to these interim results and make the final decision to terminate the trial
Harms	22	Plans for collecting, assessing, reporting, and managing solicited and spontaneously reported adverse events and other unintended effects of trial interventions or trial conduct
Auditing	23	Frequency and procedures for auditing trial conduct, if any, and whether the process will be independent from investigators and the sponsor
**Ethics and dissemination**		
Research ethics approval	24	Plans for seeking research ethics committee/institutional review board (REC/IRB) approval
Protocol amendments	25	Plans for communicating important protocol modifications (eg, changes to eligibility criteria, outcomes, analyses) to relevant parties (eg, investigators, REC/IRBs, trial participants, trial registries, journals, regulators)
Consent or assent	26a	Who will obtain informed consent or assent from potential trial participants or authorised surrogates, and how (see Item 32)
	26b	Additional consent provisions for collection and use of participant data and biological specimens in ancillary studies, if applicable *(Not Applicable)*
Confidentiality	27	How personal information about potential and enrolled participants will be collected, shared, and maintained in order to protect confidentiality before, during, and after the trial
Declaration of interests	28	Financial and other competing interests for principal investigators for the overall trial and each study site
Access to data	29	Statement of who will have access to the final trial dataset, and disclosure of contractual agreements that limit such access for investigators
Ancillary and post-trial care	30	Provisions, if any, for ancillary and post-trial care, and for compensation to those who suffer harm from trial participation
Dissemination policy	31a	Plans for investigators and sponsor to communicate trial results to participants, healthcare professionals, the public, and other relevant groups (eg, via publication, reporting in results databases, or other data sharing arrangements), including any publication restrictions
	31b	Authorship eligibility guidelines and any intended use of professional writers
	31c	Plans, if any, for granting public access to the full protocol, participant-level dataset, and statistical code

For peer review

Appendices

Informed consent materials	32	Model consent form and other related documentation given to participants and authorised surrogates
Biological specimens	33	Plans for collection, laboratory evaluation, and storage of biological specimens for genetic or molecular analysis in the current trial and for future use in ancillary studies, if applicable *(Not Applicable)*

*It is strongly recommended that this checklist be read in conjunction with the SPIRIT 2013 Explanation & Elaboration for important clarification on the items. Amendments to the protocol should be tracked and dated. The SPIRIT checklist is copyrighted by the SPIRIT Group under the Creative Commons "Attribution-NonCommercial-NoDerivs 3.0 Unported" license.

For peer review only
