# Supplementary material for: Examining the benefit of graduated compression stockings in the prevention of hospital-associated venous thromboembolism in low-risk surgical patients: a multicentre cluster randomised controlled trial (PETS trial)
Source: BMJ Open. 2023 Jan 17;13(1):e069802. doi: 10.1136/bmjopen-2022-069802 (PMC9853211; doi:10.1136/bmjopen-2022-069802)
Supplement: Reviewer comments [file bmjopen-2022-069802.reviewer_comments.pdf]

PEER REVIEW HISTORY

BMJ Open publishes all reviews undertaken for accepted manuscripts. Reviewers are asked to complete a checklist review form (**http://bmjopen.bmj.com/site/about/resources/checklist.pdf**) and are provided with free text boxes to elaborate on their assessment. These free text comments are reproduced below.

ARTICLE DETAILS

**TITLE (PROVISIONAL)**	Examining the benefit of graduated compression stockings in the prevention of hospital-associated venous thromboembolism in low-risk surgical patients: a multicentre cluster randomised controlled trial (PETS Trial)
**AUTHORS**	Machin, Matthew; Peerbux, Sarrah; Whittley, Sarah; Hunt, Beverley; Everington, Tamara; Gohel, Manjit; Norrie, John; Epstein, David; Warwick, David; Baker, Christopher; Hamady, Zaed; Smith, Sasha; Bolton, Layla; Stephens-Boal, Annya; Gray, Beverley; Shalhoub, Joseph; Davies, AH

This article was not externally reviewed at BMJ Open. Protocols that have been independently assessed prior to submission to BMJ Open are usually fast-tracked to publication on the grounds that further substantial changes will not be possible. This independent assessment will usually be external, independent review for both a substantial grant award from a non-commercial or government funder and ethics approval.
